# Assessing the gene silencing potential of AuNP-based approaches on conventional 2D cell culture versus 3D tumor spheroid

**DOI:** 10.3389/fbioe.2024.1320729

**Published:** 2024-02-12

**Authors:** Beatriz B. Oliveira, Alexandra R. Fernandes, Pedro Viana Baptista

**Affiliations:** ^1^ UCIBIO, Department Ciências da Vida, Faculdade de Ciências e Tecnologia, Universidade NOVA de Lisboa, Caparica, Portugal; ^2^ i4HB, Associate Laboratory—Institute for Health and Bioeconomy, Faculdade de Ciências e Tecnologia, Universidade NOVA de Lisboa, Caparica, Portugal

**Keywords:** gold nanoparticles, gene silencing, 3D spheroids, cancer models, c-MYC

## Abstract

Three-dimensional (3D) cell culture using tumor spheroids provides a crucial platform for replicating tissue microenvironments. However, effective gene modulation via nanoparticle-based transfection remains a challenge, often facing delivery hurdles. Gold nanoparticles (AuNPs) with their tailored synthesis and biocompatibility, have shown promising results in two-dimensional (2D) cultures, nevertheless, they still require a comprehensive evaluation before they can reach its full potential on 3D models. While 2D cultures offer simplicity and affordability, they lack physiological fidelity. In contrast, 3D spheroids better capture *in vivo* conditions, enabling the study of cell interactions and nutrient distribution. These models are essential for investigating cancer behavior, drug responses, and developmental processes. Nevertheless, transitioning from 2D to 3D models demands an understanding of altered internalization mechanisms and microenvironmental influences. This study assessed ASO-AuNP conjugates for silencing the c-MYC oncogene in 2D cultures and 3D tumor spheroids, revealing distinctions in gene silencing efficiency and highlighting the microenvironment’s impact on AuNP-mediated gene modulation. Herein, we demonstrate that increasing the number of AuNPs per cell by 2.6 times, when transitioning from a 2D cell model to a 3D spheroid, allows to attain similar silencing efficiencies. Such insights advance the development of targeted gene therapies within intricate tissue-like contexts.

## 1 Introduction

Three-dimensional (3D) cell culture relying on tumor spheroids has become a critical tool for modelling both normal and malignant tissue due to the ease of manipulation, range of possible conditions, relative low cost when compared to *in vivo* assays and capability to mimic physiological conditions. Despite the increased use of these 3D spheroids, gene modulation strategies using nanoparticle-based transfection methods have proven challenging.

There have been plenty of studies reporting on the advantages of using gold nanoparticles (AuNPs) for the transfection of gene modulating moieties into a wide range of cell models, which have relied on traditional two-dimensional (2D) cell culture approaches (Sambale et al., 2015). However, the translation of such AuNP-based approaches from conventional 2D cell culture models to the more physiologically relevant 3D tumor models, is crucial for a comprehensive evaluation of their efficacy (Da Rocha et al., 2014).

2D cultures are characterized by cell adherence and growth under the form of a monolayer on a culture flask, petri dish or attached to any other plastic surface, with considerable ease of manipulation and low-cost maintenance (Breslin and O’Driscoll, 2013). Still, they fall short of representing the real cell-cell interactions in a tumor mass (Baker and Chen, 2012; Hickman et al., 2014; Kapałczyńska et al., 2018; Juarez-Moreno et al., 2022). Furthermore, the 2D static monolayer system allows unlimited access to oxygen, nutrients, metabolites and signaling molecules, which diverge from the real conditions faced by cells on growing tumor masses.

As such, 3D *in vitro* aggregates of tumor cells, also known as spheroids, have been proposed as better models to mimic tumor behavior and microenvironment (Huh et al., 2011). This 3D arrangement of cells gets one step closer to the typical features of *in vivo* systems, such as proper cell-cell and cell-matrix interactions, creation of environmental “niches,” morphology and division preservation, diversification of phenotypes and polarity, as well as different access to oxygen, nutrients, metabolites and signaling molecules (Schmeichel and Bissell, 2003; Friedrich et al., 2009; Mehta et al., 2012). Spheroids have been used as models for colon, breast, pancreatic and other cancer diseases to aid in the discovery of new anti-cancer therapeutics, as well as in toxicological screening and developmental biology studies, offering a more representative platform for studying the new cancer therapeutical approaches in a tissue-like context (Juarez-Moreno et al., 2022).

Gene therapy approaches have been developed as tools to tackle cancer cells—from silencing specific cancer hallmarks to harnessing the immune response to tumor antigens (Mccormick, 2001). Gene silencing of activated oncogenes has been at the vanguard of these therapeutic avenues, either relying on antisense oligonucleotides (ASOs) or small interfering RNA (siRNA) (Watts and Corey, 2012). Nevertheless, its widespread application has been impaired by poor accumulation on target cells, off-target effects, and low circulating time, related to nuclease cleavage (Watts and Corey, 2012). These shortcomings highlighted the importance of a delivery system capable of transporting the ASOs to its RNA target site while preventing degradation by circulating nucleases. AuNPs-based delivery systems have been at the forefront of effective gene therapy, allowing for precise control over their synthesis and functionalization, with a high surface area-to-volume ratio, providing ample space to attach functional groups and therapeutic molecules, enhancing their stability and targeting capabilities to the desired cells or tissues (Ajnai et al., 2014; Mendes et al., 2017; Oliveira et al., 2023). Moreover, AuNPs exhibit excellent biocompatibility and low toxicity, making them suitable for *in vivo* applications (Azad et al., 1993; Watts and Corey, 2012).

**FIGURE 1:**
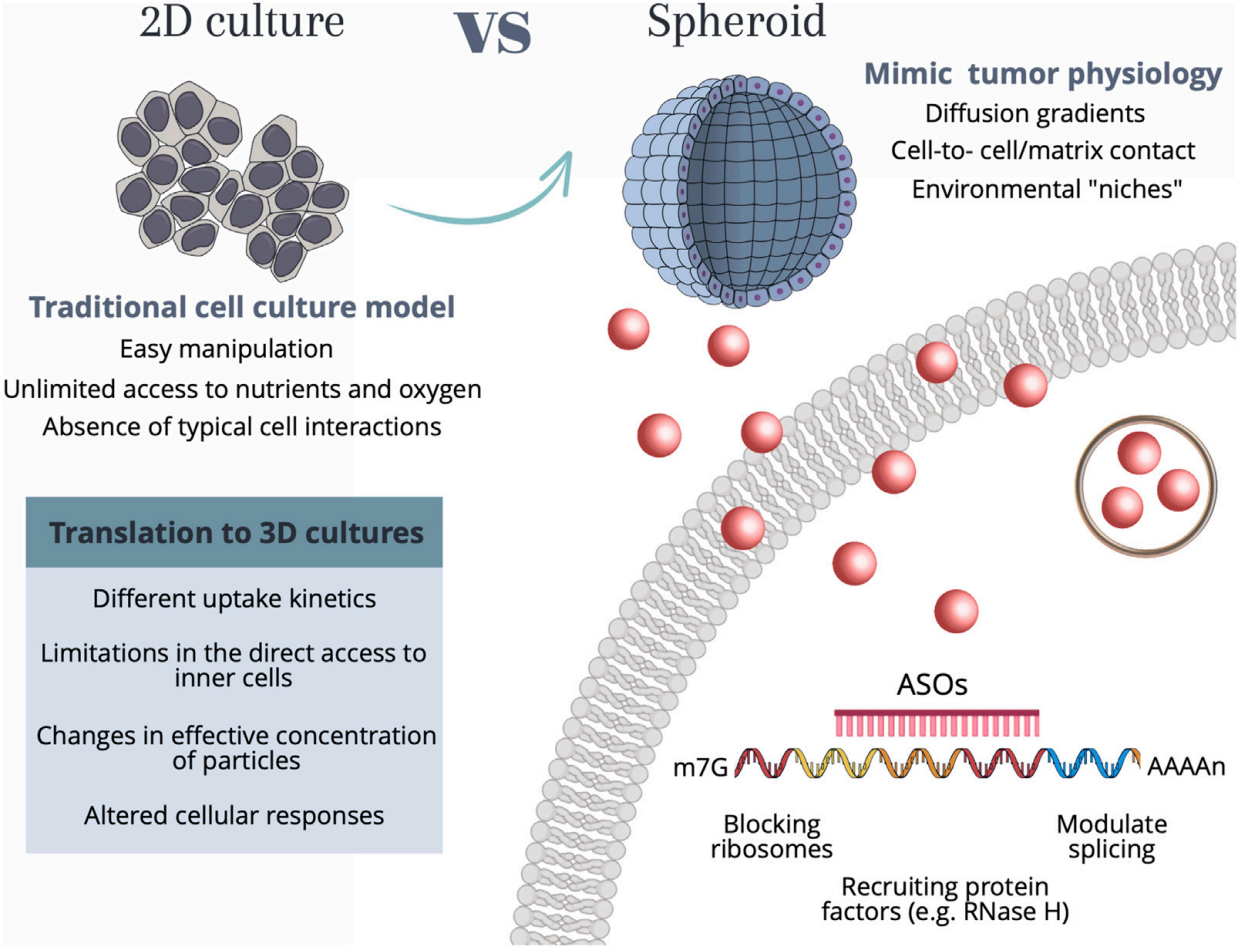
Schematic representation of the main characteristics of each cell culture model as well as the considerations required for translation between 2D culture to more complex 3D tumor spheroids. 2D cell culture methods allow for easy and cost-effective manipulation, however present shortcomings related to unlimited access to nutrients and oxygen, as well as the lack of typical cell-cell interactions. Conversely, spheroids allow for a recapitulation of the native structure of cells, by creating diffusion gradients, cell-cell interactions, and environmental “niches”, that allow cell heterogeneity. Independently of the cell culture method, the internalization of AuNPs by human cells occurs through a range of different mechanisms, among them phagocytosis, micropinocytosis, and receptor-mediated endocytosis, which use different receptors and cellular signaling pathways. These mechanisms are dependent on the size and type of nanoparticles. For AuNPs with diameters <100 nm (such as the ones used in the present work) the internalization mainly occurs by receptor-mediated endocytosis. Upon the entry of the Au-nanoconjugates in the cell, AuNPs can be arrested in vesicles such as lysosomes and further signaled for destruction, or the anti-sense DNA domain might be released and bind to the complementary mRNA site, performing the gene modulation by either blocking the ribosomes, recruiting protein factors (e.g., RNase H) or modulate the splicing. Abbreviations: m7G—7-methylguanosine (typical 5′ cap on mRNA molecules); AAAAn, poly(A) tail; ASOs, Antisense oligonucleotide sequence.

It is of the utmost importance to extend the current knowledge on nanoparticle-based gene silencing approaches supported on 2D cell cultures to the more realistic 3D spheroids. The effective translation between 2D to 3D-cell models requires a deeper understanding of the differences in the internalization mechanisms, cell-to-cell communication, extracellular matrix interactions, or diffusion gradients, that might hinder the uptake efficiency and internalization kinetics of particles, ultimately impacting the therapeutic/silencing outcome (Belli et al., 2017). Understanding these alterations is vital for successful gene silencing studies, not only improving the accuracy and relevance of experimental results but also aiding in the development of innovative strategies for targeted gene therapies in complex tissue-like environments—see Figure 1.

Herein, we compared the efficacy of ASO-AuNP conjugates to silence *c-MYC* oncogene in 2D cell models and 3D tumor spheroids. Characterization of the silencing profiles pointed out to the underlying differences in gene silencing efficiency between 2D and 3D cultures, and the impact of the cellular microenvironment, spatial organization, and cell-cell interactions on the effectiveness of gene silencing strategies utilizing AuNPs.

## 2 Materials and methods

### 2.1 Gold nanoparticle synthesis, functionalization and characterization

Gold nanoparticles with a diameter of ∼12 nm (±1) were synthesized via citrate reduction method, using 1 mM Tetrachloroauric(III) acid (HAuCl_4_·3H_2_O, 3 ≥ 49.0% Au basis; CAS: 16961-25-4, Sigma-Aldrich, United States) and 38.8 mM sodium citrate (285 mg) (HOC(COONa) (CH_2_COONa)_2_·2H_2_O ≥99.0%; CAS: 6132-04-3, Sigma-Aldrich) for a final volume of 250 mL (Conde et al., 2012a; Veigas et al., 2015). The obtained colloidal gold solution was filtered through a 0.2 um filter membrane (Pall Acrodisc™ 32 mm Syringe Filter with 0.2 um Supor™ Membrane, REF 4645, Pall, United States) and stored at room temperature protected from light. The AuNP concentration was determined by measuring the absorption and assuming a molar absorptivity for the plasmon resonance band maximum (520 nm) of 2.33 × 10^8^ M^−1^cm^−1^ (Liu et al., 2007).

AuNPs were subsequently functionalized with thiol-modified polyethylene glycol (PEG) to attain 30% of coverage. Briefly, 10 nM of citrate-capped AuNPs were incubated with 0.028% (w/v) SDS (Sodium dodecyl sulphate) (CAS: 151-21-3, Sigma-Aldrich), and 0.003 mg/mL of PEG (O-(2-Mercaptoethyl)-O′-methyl-hexa(ethylene glycol) (Ref 672572, Sigma-Aldrich), for a period of 16 h under agitation (Pedrosa et al., 2018). The unbound PEG molecules were removed though two centrifugations of 45 min at 14,000 xg and 4°C. The functionalization efficiency was assessed with Ellman’s assay [5,5-dithio-bis-(2-nitrobenzoic acid, Cat. No. 22582, Thermo Scientific™, United States].

AuNPs@30%PEG were further functionalized with thiolated oligonucleotides (5′-thiol-(CH2)6-ssDNA oligo) (STAB Vida, Lda., Portugal). For the specific silencing of *c-MYC* gene (NCBI Reference Sequence: NG_007161.2), a sequence 5′-thiol- GCG CCC ATT TCT TCC AGA TAT CCT CGC TGG GCG C-3′ was used [AuNP@c-MYC] (Vinhas et al., 2018) Additionally, a scramble control, with a sequence without any target on the human genome was also functionalized on the particles 5′-thiol-TT CGG GTT GAC GTT AGC CGG ATC TAC CGA AA-3′ [AuNP@Scramble] (Vinhas et al., 2018). The referred sequences were added to the AuNPs@30%PEG in a ratio of 1:150 (AuNP:oligonucleotide), after thiol reduction with 0.1 M of DTT and subsequent purification using a NAP-5 desalting column, according to the manufacturer’s instructions. After incubation of the AuNPs with the purified oligonucleotides, the ionic strength of the solution was gradually increased. As so, the Au-nanoconjugates were firstly incubated for 20 min with 10 mM phosphate buffer pH = 8, 2% SDS (AGEI), to a final concentration of 10 mM phosphate buffer (pH = 8) and 0.01% (w/v) SDS. Then, 10 mM phosphate buffer pH = 8, 1.5 M NaCl, 0.01% SDS (AGE II) was added in appropriate volumes to a final concentration of 10 mM phosphate buffer (pH = 8), 0.05 M NaCl and 0.01% (w/v) SDS. Serial additions of AGE II were performed to attain final concentrations of 0.1, 0.2 and 0.3 M of NaCl. Upon each AGE addition, a step of 10 s of ultrasounds and 20 min incubation under agitation were performed. The final solution was incubated in the dark for 16 h at room temperature under agitation. The excess oligonucleotides were removed by two centrifugations of 1 h at 15,500xg, the supernatants were recovered, and the amount of oligonucleotide quantified using a NanoDrop (Nanodrop 2000 Spectrophotometer, Cat# ND-2000, Thermo Scientific). The number of ASOs bonded to the AuNPs’ surface was estimated by subtracting the number of ASOs present in the supernatants recovered from the NPs washes to the initial amount of ASOs incubated with NPs (Conde et al., 2013; Baptista et al., 2013; Sousa and Conde, 2022).

All gold nanoparticles and nanoconjugates (AuNP@Citrate, AuNP@30%PEG, AuNP@c-MYC and AuNP@Scramble) were analyzed by UV-vis spectroscopy (UVmini 1240, Shimadzu, JP), electrophoresis on agarose gel, dynamic light scattering for hydrodynamic size and zeta potential with a Malvern Zetasizer (Malvern Panalytical, MAL1210370, United Kingdom) and by transmission electron microscopy (TEM) (JEOL, 1200EX electron microscope, United States) (Susnik et al., 2023) (Supplementary Figure S1). Additionally, the stability of the Au-nanoconjugates in the cell medium was also assessed by Dynamic Light Scattering (DLS) and UV-vis spectroscopy (Supplementary Figure S2).

### 2.2 Cell culture and maintenance and spheroid preparation

HCT-116 (ATCC^®^ CCL-247™) tumor cell line purchased from American Type Culture Collection (ATCC^®^, United States) was grown in Dulbecco’s Modified Eagle Medium (DMEM; Invitrogen, United States) and supplemented with 10% (v/v) Fetal Bovine Serum (FBS; Invitrogen, United States) and 1% (v/v) antibiotic/antimycotic (Invitrogen, United States) and maintained in 25 cm^2^ culture flasks (VWR, United States) at 37°C in a 99% humidified atmosphere of 5% (v/v) CO_2_ (CO_2_ Incubator Leec, United Kingdom).

HCT-116 spheroids were prepared according to Baek et al. (Baek et al., 2016; Roma-Rodrigues et al., 2020). Cells were seeded at a density of 5 × 10^3^ cells per well in a super-low attachment U-shape 96-well culture plate (BIOFLOAT™ 96-well plate, faCellitate, DE), and grown for 3 days, obtaining spheroids with a 570 µm (±3) diameter (Supplementary Figure S3).

### 2.3 Challenge of cells

Cells were incubated with the 3 different nanoconjugates: AuNP@c-MYC for specific silencing of the *c-MYC* oncogene; AuNP@Scramble functionalized with a control sequence not recognizing any target within the cells, and AuNP@PEG that serves as control for the impact of NP uptake into cells.

The 2D and 3D cells were challenged at varying concentrations of oligonucleotide: 20nM, 30nM, 33nM, 40nM, 54nM, 70nM and 88 nM, corresponding to 0.17nM, 0.25nM, 0.28nM, 0.34nM, 0.45nM, 0.59nM and 0.74 nM of gold, respectively; and at different incubation times: 3h, 6h, 12h, 18h and 24 h. The AuNP@PEG control was performed simultaneously so that the concentration of gold matches the one used for AuNP@c-MYC [AuNP@PEG M] or AuNP@Scramble [AuNP@PEG S]. Additionally, controls for 2D-cells or 3D spheroids alone were performed, where only DMEM medium was added to the wells.

For 2D-cell challenge assays, HCT-116 cells were seeded at a density of 1 × 10^5^ cells per well in a 24-well plate (Cat. No. 30024, SPL Life Sciences, KR) and incubated for 24 h on a CO_2_ incubator to allow cell adherence. For 3D-cell challenge assays, spheroids previously seeded at a density of 5 × 10^3^ cells per well were used on the third day of growth. Before incubating the cells/spheroids with the nanoconjugates, the medium was removed from the wells and replaced by a solution with medium and the respective nanoconjugate at the referred concentrations. After the designated period of incubation, the supernatant was removed, and the cells detached using TrypLE Express (TrypLE Express reagent, Gibco, United States) and further centrifuged for 5 min at 500xg (2D cells) or 5 min at 1000xg (spheroids). The obtained pellet was either used for RNA/protein extraction or inductively coupled plasma atomic emission spectroscopy (ICP-AES).

### 2.4 Gene expression

For RNA extraction of 2D-cell assays, 2 wells (of 24-well plates) were used for each condition, whereas, for the 3D-cultures, a total of 8 spheroids were used. RNA was extracted using NZYol reagent (MB18501, NZYTech, PT), and following the manufacturer’s specifications After extraction, the integrity of the RNA was assessed by 1% agarose gel electrophoresis (Agarose with electrophoresis grade, MB02703; NZYTech) and the RNA concentration quantified using a NanoDrop. Next, the RNA was diluted to 10ng/uL, from which 1 uL was used as template for the 1-step RT-qPCR reaction, using the One-step NZYSpeedy RT-qPCR Green kit (NZYTech, PT). A 229-base pair (bp) fragment of the human *c-MYC* oncogene (Ac. No. NM_002467) and a 215 bp fragment of the human *18S* N5 ribosomal RNA (Ac. No. NR_003286) were amplified using the following primers: *c-MYC* Forward 5′-TCT​GAA​GAG​GAC​TTG​TTG​C-3′ and *c-MYC* Reverse 5′-TTC​AGT​CTC​AAG​ACT​CAG​C-3’; *18S* Forward 5′-AGG​GTT​CGA​TTC​CGG​AGA​G-3′ and *18S* Reverse 5′-GAA​TTA​CCG​CGG​CTG​CTG-3’. The reaction mixture was prepared following the manufacturer’s specificities, containing 400 nM of each primer, 1x One-step NZYSpeedy qPCR Green master mix, 0.4 uL of NZYRT mix, 10 ng of RNA and DEPC-treated water up to 10 uL. The amplification was conducted on a Rotor-Gene Q 5plex HRM Platform (Cat. No. 9001580, Qiagen, GE), with the following thermal conditions: 50°C for 20 min (Reverse transcription step), 95°C for 3 min (Denaturation step) followed by 40 cycles of 95°C for 30 s and 60°C for 50 s. The obtained PCR products were then analyzed on a 1.5% agarose gel electrophoresis stained with 1x GelRed (Biotium, United States).

The silencing potential of the anti-*c-MYC* Au-nanoconjugate was assessed via the 2^−ΔΔCT^ method. For each experience both *c-MYC* (target gene) and *18S* (reference gene) were amplified, and the respective Cycle Threshold (CT) values were acquired using the Corbett Rotor-Gene 6000 version 1.7 Software, setting a threshold of 0.0015. The delta CT (△CT) was calculated for each control (CT_c-MYC_—CT_18S_), and delta delta CT (△△CT) was calculated by subtracting the △CT to the respective control (Livak and Schmittgen, 2001; Schmittgen and Livak, 2008). Relative Change values were calculated to highlight the effect of the anti-c-MYC ASOs sequence. As such, a ratio between of the 2^−ΔΔCT^ of the interest sample with the respective control was performed. For the complete formula see Supplementary Material S4. The results were analyzed using one-way ANOVA (Kruskal–Wallis test) and non-parametric *t*-test (Mann-Whitney test) with GraphPad Prism Version 8.0.1 (244) software. Bars on graphs represent the average result of at least 3 biological replicates with 3 technical replicates and error bars the respective Standard Error Mean (SEM) value or Standard Deviation.

### 2.5 Cell viability

Cytotoxicity of the Au-nanoconjugates on the 2D-cell system was assessed by the MTS assay (Promega Corporation, United States). Briefly, cells were seeded at a density of 0.75 × 10^4^ cells/well (96-well plate), and after the cell exposure to the nanoconjugates, the MTS reagent was diluted in culture media (1:5) and added to the cells. After 45 min of incubation on a CO_2_ Incubator at 37°C, the absorbance at 490 nm was measured on a microplate reader (Infinite^®^ 200 PRO, Tecan, SZ) (Fernandes et al., 2017).

For the assessment of cell viability on spheroids, the CellTox™ Green cytotoxicity assay (Promega) was used following the manufacturer’s recommendations. Briefly, following spheroid incubation with the Au-nanoconjugates, culture medium was removed and replaced by CellTox™ Green dye 1x prepared on DMEM medium, without phenol red and incubated for 24 h (Chiaraviglio and Kirby, 2014; Roma-Rodrigues et al., 2020). Images of the spheroids were acquired with a Ti-U eclipse inverted microscope (Nikon, Tokyo, Japan) and respective software NIS Elements Basic software vs. 3.1 (Nikon, Tokyo, Japan), using the Fluorescein isothiocyanate (FITC) filter (excitation at 480/30 nm and emission at 535/45 nm) with 800 ms of exposure time. For 100% of cell death control, spheroids were fixated with 4% paraformaldehyde solution in PBS, following the PFA addition spheroids were incubated for 20 min at room temperature. The ImageJ software (https://imagej.net/, version 1.53a) was used to measure the green fluorescence intensity (mean fluorescence x area) of the total spheroid, by delimiting its periphery. The fluorescence intensity was normalized to the fluorescence of the background (of the correspondent image). The final cell viability was attained by normalizing the corrected fluorescence intensity of each condition to the 100% cell death control (spheroids fixated with PFA). Bars in graphs represent the average of ten different spheroids, and error bars the respective standard deviation.

### 2.6 Protein expression—Western blot

Total protein was extracted from HCT-116 cells, either seeded in 24-well plate (2 wells per condition) or from spheroids (10 spheroids per condition). The 2D-cells were detached with Triple Express, whereas the spheroids were simply recovered from the wells, the pellet was then washed with PBS 1x and resuspended in lysis buffer [150 mM NaCl, 50 mM Tris (pH 8.0), 5 mM EDTA, 2% (v/v) NP-40, 1× phosphatase inhibitor (PhosStop, Roche), 1× protease inhibitor (cOmplete Mini, Roche), 1 mM PMSF, and 0.1% (w/v) DTT] and incubated overnight at −80°C. Then, the whole-cell extracts were sonicated following a sequential increase in intensity: 10 pulses at 60% intensity (repeat 5 times), 15 pulses at 70% intensity (repeat 15 times), and 10 pulses at 80% intensity (repeat 10 times). Following sonication, extracts were centrifuged at 10,000 × g for 5 min at 4°C. The supernatant was recovered, and protein concentration was determined using the Pierce 660 nm Protein Assay Reagent (ThermoFisher Scientific) following the manufacturer’s specifications. Then, 20 μg total protein extracts were separated by SDS-PAGE in a 5% acrylamide gel (Merck Millipore). The electrophoretic transfer was performed using the semi-dry transfer method (Cleaver Scientific, United Kingdom) for 1 h at 120 mA. Proteins were transferred onto a 0.45 μm polyvinylidene difluoride (PVDF) membrane (Amersham Hybond 0.45 μm PVDF; Amersham™, United Kingdom), further blocking was performed for 2 h at room temperature with 5% (w/v) milk solution in Tris-buffered saline with 0.1% (v/v) Tween 20 (TBST). The incubation of membranes with antibodies was performed following the manufacturer’s instructions: overnight incubation at 4°C with the primary antibody against c-MYC (reference no. ab32072, Abcam) dilution 1:1000 in the blocking solution, and 1 h at room temperature for β-actin (for normalization purposes; reference no. A5441, Sigma) (dilution 1:5000). Membranes were washed with TBST and incubated with the appropriate secondary antibody conjugated with horseradish peroxidase (reference no. 7074 (1:2000) or 7076 (1:3000), Cell Signaling Technology). WesternBright ECL (Advansta) was applied to the membranes, and signal was acquired on a dark room using a Hyperfilm™ ECL™ film (GE Healthcare), with 1 min of exposure time for C-MYC protein and 15 s for β-actin.

### 2.7 Nanoparticle uptake

The gold content in cells was assessed by ICP-AES to provide information on nanoconjugates uptake. AuNP@c-MYC nanoconjugates were incubated with HCT-116 cells in 2D culture (54nM and 88 nM) and spheroids (54nM and 33 nM) for 3h, 6h, 12h, 18h and 24 h. After incubation the supernatants were recovered, and cells were detached from the wells as performed for RNA extraction. For spheroids, a 5 min centrifugation at 1000 *g* was performed to separate the spheroids from the medium. Cellular pellets and supernatants were incubated overnight with *aqua regia*. Samples were analyzed by ICP-AES to assess the Au content (paid service performed on Laboratório de Análises from the Chemistry Department at NOVA University). This allowed to estimate the gold content per cell. A total of 4 wells (24-well plate) and 20 spheroids were used per condition for the 2D and 3D cultures, respectively.

## 3 Results and discussion

The use of 3D cell models for the evaluation of gene silencing strategies vectorized by nanoparticles is a promising technique with potential applications in models for studying gene function, understanding disease mechanisms, and developing targeted therapies. In fact, 3D and 2D cell models are both important tools in cell biology and biomedical research, but they each have distinct advantages and applications depending on the research objectives. Of extreme relevance is the possibility of easily translating findings from traditional 2D models towards increasingly complex 3D cell models, which can provide more accurate representations of disease states and better assess the potential of new treatments. There are several challenges to overcome when translating data of gene silencing via nanoparticles from 2D to 3D cell models, such as the need to optimize delivery efficiency, ensure specific and efficient targeting of genes, and address potential off-target effects, which is of paramount relevance to ensure comparison between systems.

Herein, we used gold nanoconjugates targeting the *c-MYC* oncogene in both 2D and 3D cell models of colorectal cancer to calibrate the efficiency of silencing potential upon the increase in cellular complexity (see Figure 1).

### 3.1 Characterization of gold nanoconjugates

**FIGURE 2:**
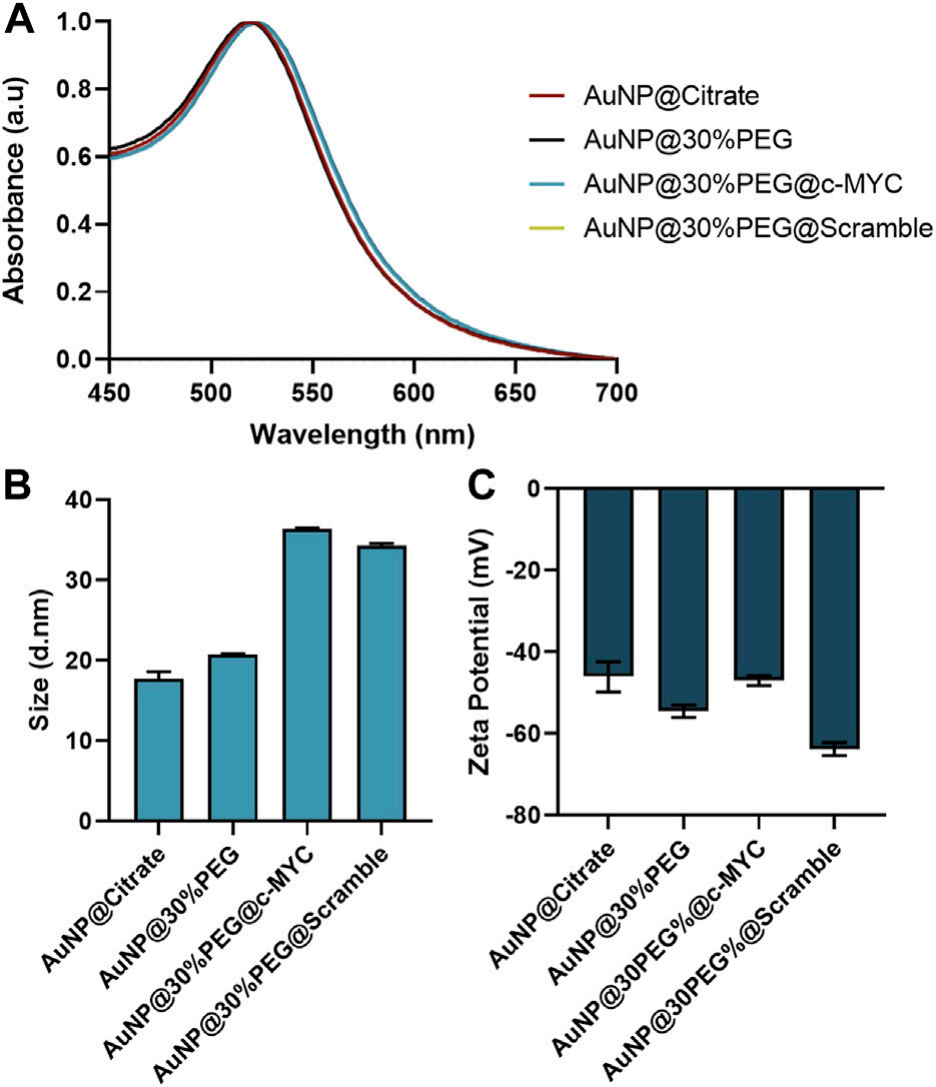
Characterization of Au-nanoconjugates by UV-Vis spectroscopy, DLS, and Zeta-potential. **(A)** Normalized UV-vis spectroscopy results. All nanoconjugates show the maximum peak correspondent to the Surface Plasmon Resonance (SPR) peak at 520 ± 2 nm. **(B)** Dynamic Light Scattering (DLS) results for the different nanoconjugates. **(C)** Zeta potential results for the different nanoconjugates. The error bars represent the standard deviation for 3 different batches of particles, each with 3 independent measurements.

The synthesized citrate capped-AuNPs were conjugated with PEG to a 30% of surface coverage, to provide increased biocompatibility and stability, while allowing for further conjugation with the desired ASO (Conde et al., 2013; Mendes et al., 2017; Vinhas et al., 2017; Roma-Rodrigues et al., 2020; Oliveira et al., 2020). For specific silencing, a thiolated-ssDNA oligonucleotide harboring a sequence targeting a region of the *c-MYC* oncogene was functionalized onto the AuNPs@30%PEG attaining a final functionalization of ∼100 chains of oligo per particle (Supplementary Figure S1). Additionally, a scramble (without sequence homology in the human genome) ssDNA oligonucleotide was also used as a control. Each step of the conjugation was monitored by DLS, Zeta potential measurements, and UV-Vis spectroscopy (Figure 2).

Upon each step of functionalization, the surface plasmon resonance (SPR) peak suffers a slight red-shift (Figure 2A), indicating modifications at the surface of the AuNPs (Cabral and Baptista, 2014), which are corroborated by the slight increase to the hydrodynamic diameter of the nanoconjugates due to the molecules conjugated to their surface: AuNP@Citrate (17.83 ± 0.18 nm) < AuNP@30%PEG (20.74 ± 0.18 nm) < AuNP@30%PEG@c-MYC (36.58 ± 0.16 nm) ∼ AuNP@30%PEG@Scramble (34.28 ± 0.17 nm) (Figure 2B). Additionally, the overall surface charge of the particles decreased when measured by Zeta potential, also indicating the binding of the oligonucleotides to the particles’ surface (Figure 2C). Furthermore, the functionalization was also monitored via an agarose gel electrophoresis (Supplementary Figure S1F). The different electrophoretic mobility indicates the presence of oligonucleotides on the AuNP@c-MYC and AuNP@Scramble nanoconjugates, noticeable by the higher migration towards the positive electrode. For AuNPs@30%PEG, migration was much less, since these nanoconjugates exhibit more neutral surface charge (as shown by the Zeta potential measurements) (Zhang et al., 2014). Altogether these results show the successful functionalization of the AuNPs with equivalent physical features. Finally, all the nanoconjugates show strong surface charge (−50 to −68 mV), which has been reported as a good indicator for the stability of the colloidal solution (Doane et al., 2012; Pan et al., 2012; Watters et al., 2012; Gumustas et al., 2017) (see Stability studies of each nanoconjugate in Supplementary Figure S2).

### 3.2 *c-MYC* gene silencing in 2D vs. 3D cell models

The silencing potential of the gold formulations was firstly assessed in a 2D colorectal cell model by challenging cells for 6 h with different concentrations of the anti-c-MYC ASO, ranging between 20 nM to 70 nM, corresponding to 0.17 nM–0.60 nM of gold nanoconjugates, respectively (Persengiev et al., 2004; Watts and Corey, 2012; Vinhas et al., 2017; Vinhas et al., 2018). This allowed us to identify the most effective concentration of Au-nanoconjugate for this model since the literature regarding gene silencing strategies based on ASOs and siRNA has reported the use of concentration of interfering oligonucleotides between 20 nM to 200 nM.

**FIGURE 3:**
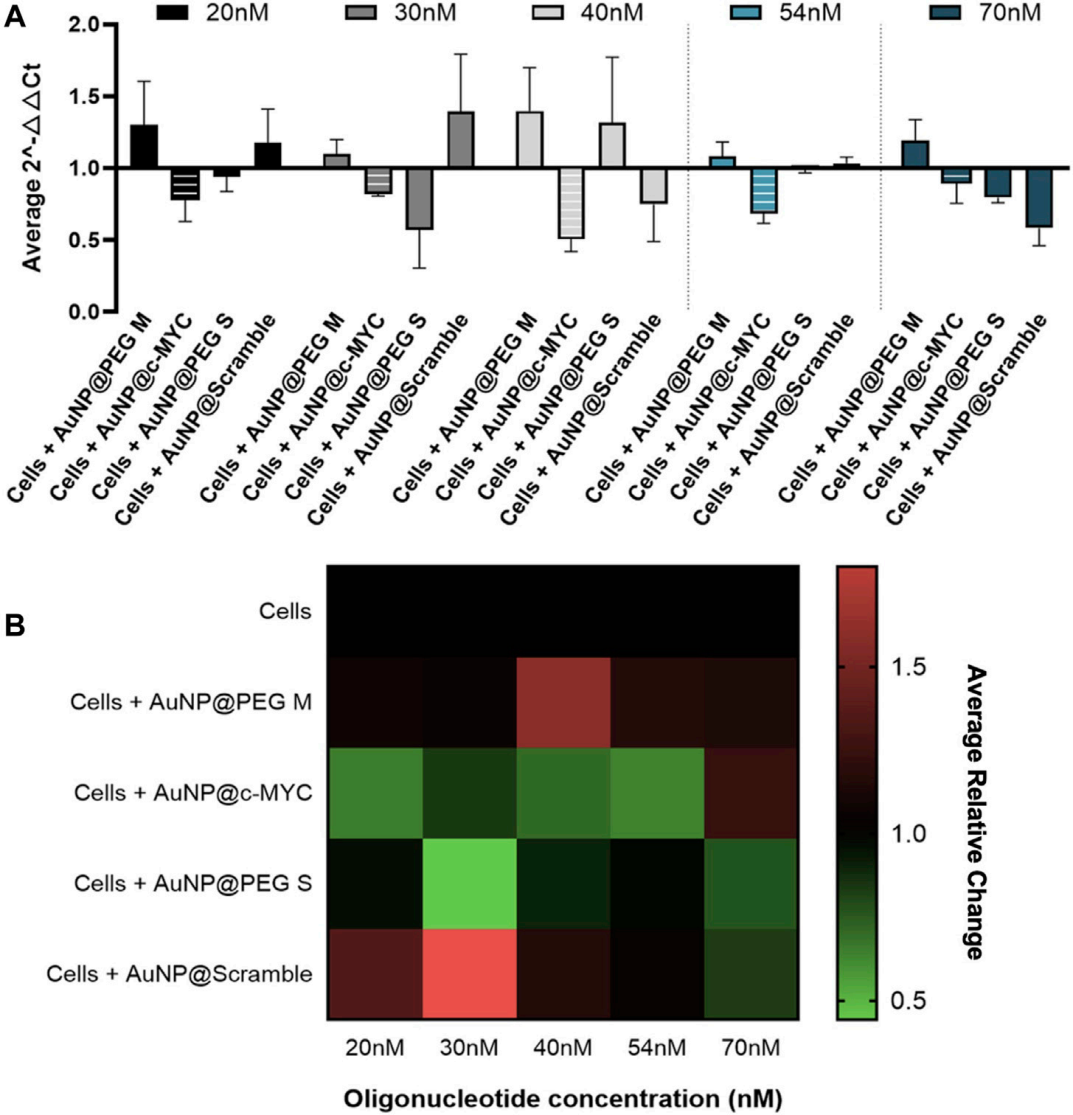
2^-△△CT^ and Relative Change results of *c-MYC* silencing using concentrations of anti-c-MYC oligonucleotide ranging between 20nM to 70 nM for 6 h of incubation. **(A)** Average 2^-△△CT^ results of *c-MYC* silencing. Full bars represent the experiment controls for cells incubated with (medium only, AuNPs@30%PEG M/S and AuNPs@Scramble), while striped bars represent the result for the cells challenged with AuNP@c-MYC ASO. Cells were incubated with the respective AuNP-oligo at a concentration of 20 nM (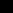), 30 nM (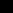), 40 nM (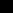), 54 nM (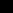) and 70 nM (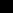). Bars are the result of 3 independent biological replicates and the error bars are the respective Standard deviation. **(B)** Average relative change results of *c-MYC* silencing. Each line represents the different AuNP-oligo controls, and the columns the concentration at which each ASO was incubated in the cells. Relative change results between 0.5 and 1 (gene silencing) are represented by green tones (from brighter to darker shades), relative change values equal to 1 are represented in black, and values between 1 and 1.8 (gene overexpression) are represented in red tones (from darker to brighter). Relative change values are the average result of 3 independent biological replicates.

All the concentrations of AuNP@c-MYC had a silencing effect on c-MYC gene when analyzed by 2^-△△CT^ method (Figure 3A). Nevertheless, no correlation between the concentration of Au-oligonucleotide conjugate and the resulting gene silencing was demonstrated, which may be attributed to several factors, among them the turnover time of the mRNA molecules and the concentration dependence of cellular uptake and intracellular trafficking mechanisms, possibly leading to variations in the nanoconjugates’ distribution within the cell, which might result in different localization patterns that do not necessarily enhance the interaction with the target mRNA molecule (Shukla et al., 2005; Alabi et al., 2013; Fernandes et al., 2017). Furthermore, the intracellular processing mechanisms of Au-nanoconjugates might not scale linearly with the dose, and the effect of nanoparticles on the regulatory networks of genes related to cell proliferation and growth (e.g., *c-MYC*) (Marano et al., 2011; Chueh et al., 2014; Li and Tang, 2020). As such, the ideal concentration of Au-oligonucleotide conjugate should be the one that only shows silencing with the AuNP@c-MYC (2^-△△CT^ < 1), while the controls cause no effect on cells (2^-△△CT^ ≥ 1). The decrease in expression of *c-MYC* gene is highlighted by the relative change shown in the heatmap (Figure 3B). Even though the most promising silencing conditions seem to be the 20nM and 54 nM oligo (21% and 28% of silencing, respectively), a more pronounced interference seems to be occurring for the controls associated with the 20 nM nanoformulation. As such, the concentration of 54 nM was selected for subsequent studies (Watts and Corey, 2012).

The cell toxicity of these Au-nanoformulations was also assessed via the MTS assay (Supplementary Figure S4A). Results show no cytotoxicity for the range of concentrations used (0.17nM, 0.25nM, 0.34nM, 0.45nM, and 0.59 nM of gold, corresponding to the oligo concentrations used for gene silencing experiments).

This is in line with the available information highlighting the biocompatibility and absence of acute toxic effects of AuNPs, either using *in vitro* or *in vivo* models (Connor et al., 2005; Shukla et al., 2005; Rosi et al., 2006; Khan et al., 2007; Trickler et al., 2011; Rambanapasi et al., 2016; Senut et al., 2016). Nevertheless, it is important to note that the cytotoxicity effects of AuNPs depend on size, shape, coating agents, dose used as well as the number of cells exposed at a given concentration, which is not always reported (Alkilany and Murphy, 2010).

Taking the AuNP@c-MYC at 54 nM concentration, exposure time profiles were also assessed for 3, 6, 12, 18, and 24 h (Supplementary Figure S5). Data show a pronounced *c-MYC* downregulation when cells are exposed to 54 nM of AuNP@c-MYC for 6 and 12 h, attaining a silencing effect of 33% and 23%, respectively.

A cell viability evaluation for these conditions was also performed (Supplementary Figure S4B). Results show that between 3 to 12 h of cell exposure, no significant effect in 2D cell viability is observed (ISO, 2009). However, for 18 h cell viability showed a slight increase, which might be associated with increased mitochondria activity resulting from cellular stress responses or due to the cell duplication rate (Tournebize et al., 2013). At 24 h, a decrease to cell viability is observable, indicating that long exposure times of cells to nanoconjugates might be causing some cytotoxicity (Rosi et al., 2006); (Trickler et al., 2011); (Rosi et al., 2006).

Considering these initial calibration studies, the best incubation condition was set for 6 h with 54 nM of AuNP@c-MYC for a 37% silencing of the *c-MYC* oncogene (Supplementary Figure S6) (Shen et al., 2005; Conde et al., 2012b; Zhao et al., 2013; Gill et al., 2021).

The translation of gene silencing via nanoparticles from 2D to 3D-cell models relies on adjusting conditions to consider differences in AuNP internalization and diffusion into the 3D structure, which play an important role in dictating silencing efficacy. In 2D-cell cultures, AuNPs passively diffuse onto cells, whereas in 3D-cell models, the architecture and organization in the three-dimensional matrix presents additional challenges, i.e., cells must penetrate multiple cell layers to reach the target (Goodman et al., 2008; Bhise et al., 2010; Van Zundert et al., 2020). This usually involves adjusting dose and incubation time of administration/challenge.

**FIGURE 4:**
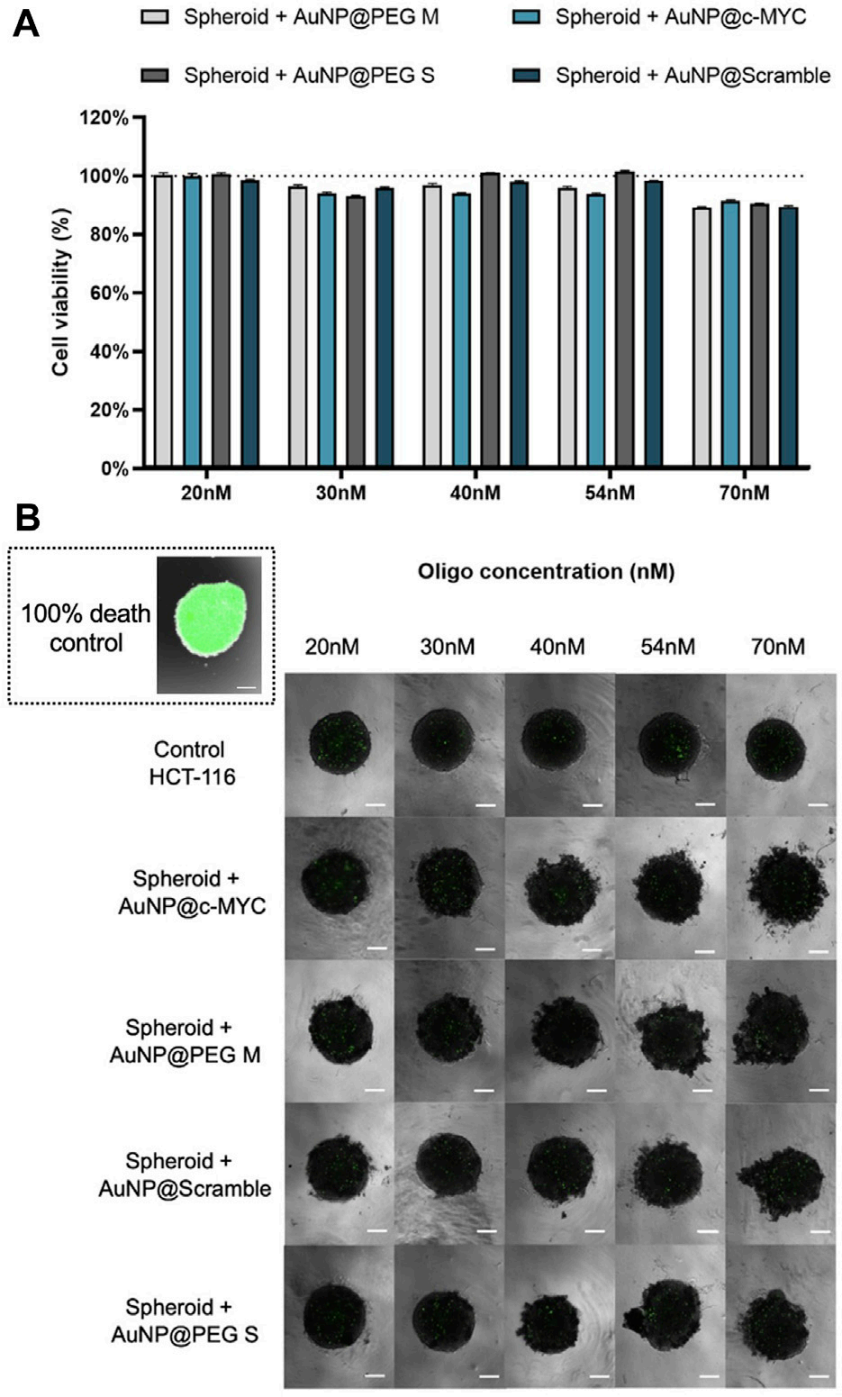
Cell viability of spheroids after 6 h of challenge with each nanoconjugate. **(A)** Bars represent the normalized cell viability when spheroids are incubated for 6 h with (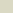) AuNP@30%PEG M, (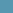) AuNP@c-MYC, (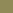) AuNP@PEG S and (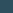) AuNP@Scramble. at different concentrations: 20nM, 30nM, 40nM, 54nM and 70 nM. Bars are the average result of 10 independent spheroids and the error bars are the respective Standard deviation. The results were obtained by measuring the green fluorescence intensity (mean fluorescence x area) of the total spheroid, by delimiting its periphery, and then normalized to the fluorescence of the background (of the correspondent image). The final cell viability was attained by normalizing the corrected fluorescence intensity of each condition to the 100% cell death control (spheroids fixated with PFA). **(B)** Spheroids stained with CellTox™ Green dye after incubation with different concentrations of each nanoprobe. The spheroid on the inset represents a typical 100% cell death control, attained after fixation with PFA. Composite image obtained through the overlap of the brightfield image with the green channel using the ×4 objective, followed by only the image attained with the green channel. All the images contain a scale bar of 100 μm.

Firstly, we evaluated the cytotoxic effect on the spheroids for the range of concentrations of Au-nanoconjugates between 20 nM to 70 nM (Figure 4).

As for the 2D model, the nanoconjugates show no toxicity effect on cells. However, some spheroid disintegration is observed upon particle incubation, which may be related to disruption of cell-cell interactions by AuNPs. It has been shown that, when interacting with cell surface proteins or integrins, AuNPs may interfere with these intercellular connections, impacting the stability of spheroids (62).

**FIGURE 5:**
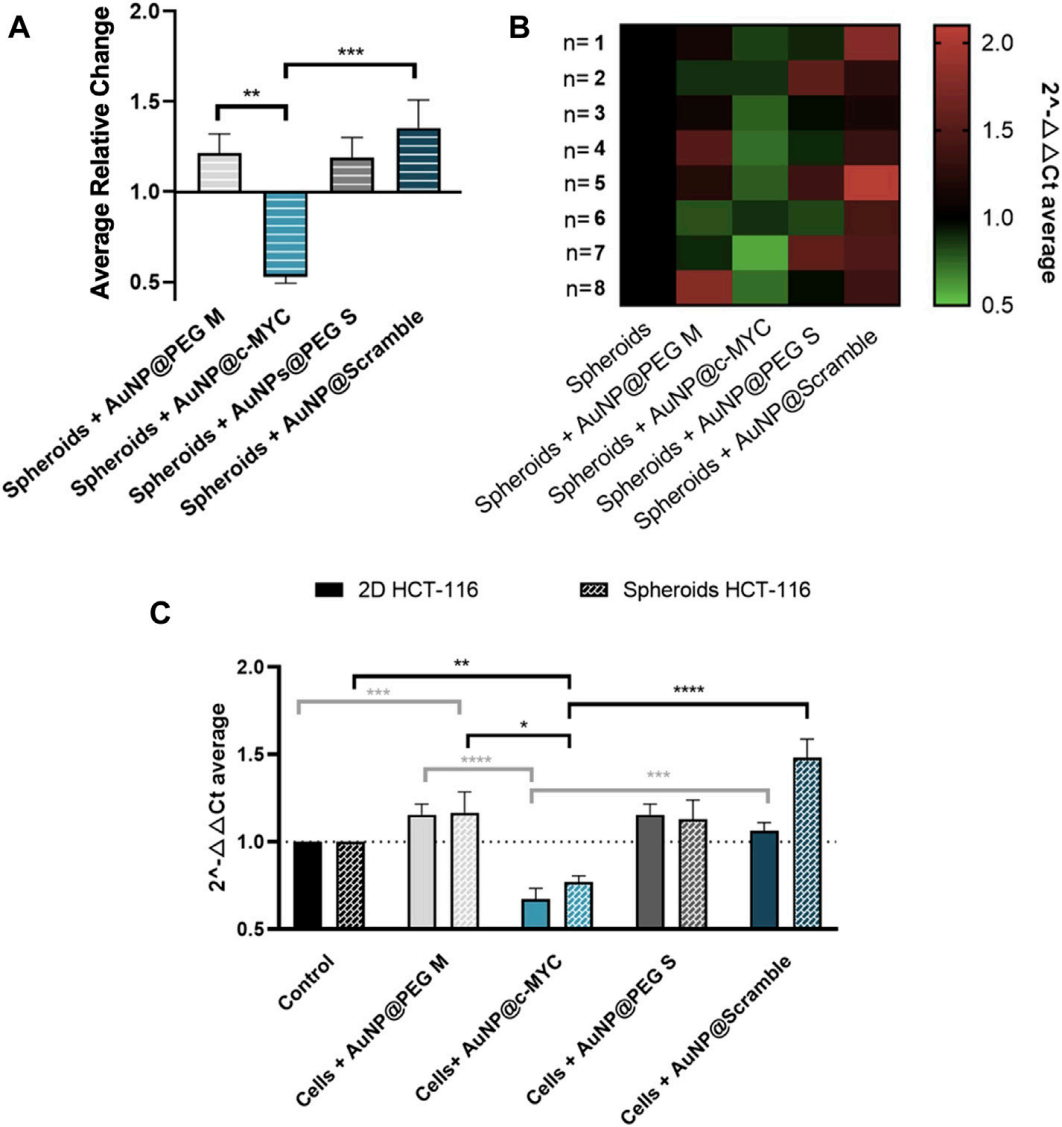
Results of *c-MYC* silencing on HCT-116 spheroids upon 6h of incubation with 54 nM of oligonucleotide. **(A)** Average relative change and **(B)** Heat-map representation of the average 2^-△△CT^ results of *c-MYC* silencing on spheroids. Each column represents the different Au-oligonucleotide conjugates controls, and the lines the results of each biological replicate. 2^-△△CT^ values between 0.5 and 1 (gene silencing) are represented by green tones (from brighter to darker shades), 2^-△△CT^ values equal to 1 are represented in black and values between 1 and 2 (gene overexpression) are represented in red tones (from darker to brighter). **(C)** Comparison between the silencing results on 2D and 3D-cell culture models. Full bars represent the 2^-△△CT^ values obtained for 2D-cell culture and the dotted bars the spheroids, for each control: (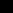) Cells incubated with medium only, (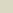) AuNPs@30%PEG M, (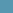) AuNP@c-MYC, (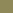) AuNPs@PEG S and (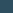) AuNPs@Scramble. Bars are the result of 8 independent biological replicates and the error bars are the respective Standard Error Mean. Statistical analysis was performed using One-way ANOVA and Mann-Whitney test, results were considered statistically significant for *p* values <0.05. (*) represents *p* < 0.0323, (**) represents *p* < 0.0021, (***) represents *p* < 0.0002 and (****) represents *p* ≤ 0.0001.

Then, the level of *c-MYC* silencing for the conditions previously set for 2D-cell culture model was evaluated - Figure 5 (see also Supplementary Figure S7 for silencing results with other incubation times). Data show a robust silencing effect of 33%, observable across 8 independent biological replicates (Figures 5A, B), similar to that attained for the 2D-cell model (37%) (panel C).

### 3.3 c-MYC protein silencing

Next, the silencing effect of the developed nanoprobe in combination with the chosen conditions was also studied at the protein level (see Supplementary Figure S8). The results show that not only the silencing effect occurs at the mRNA level (34% - relative change), but it also translates into the protein level, causing a 43% decrease in the c-MYC protein expression.

Similarly, in spheroids, the AuNP@c-MYC also reduced expression of the target protein by 47% (see Supplementary Figure S9), corresponding to the same silencing efficiency observed on the mRNA level (Supplementary Figure S9C)—considering the relative change values.

**FIGURE 6:**
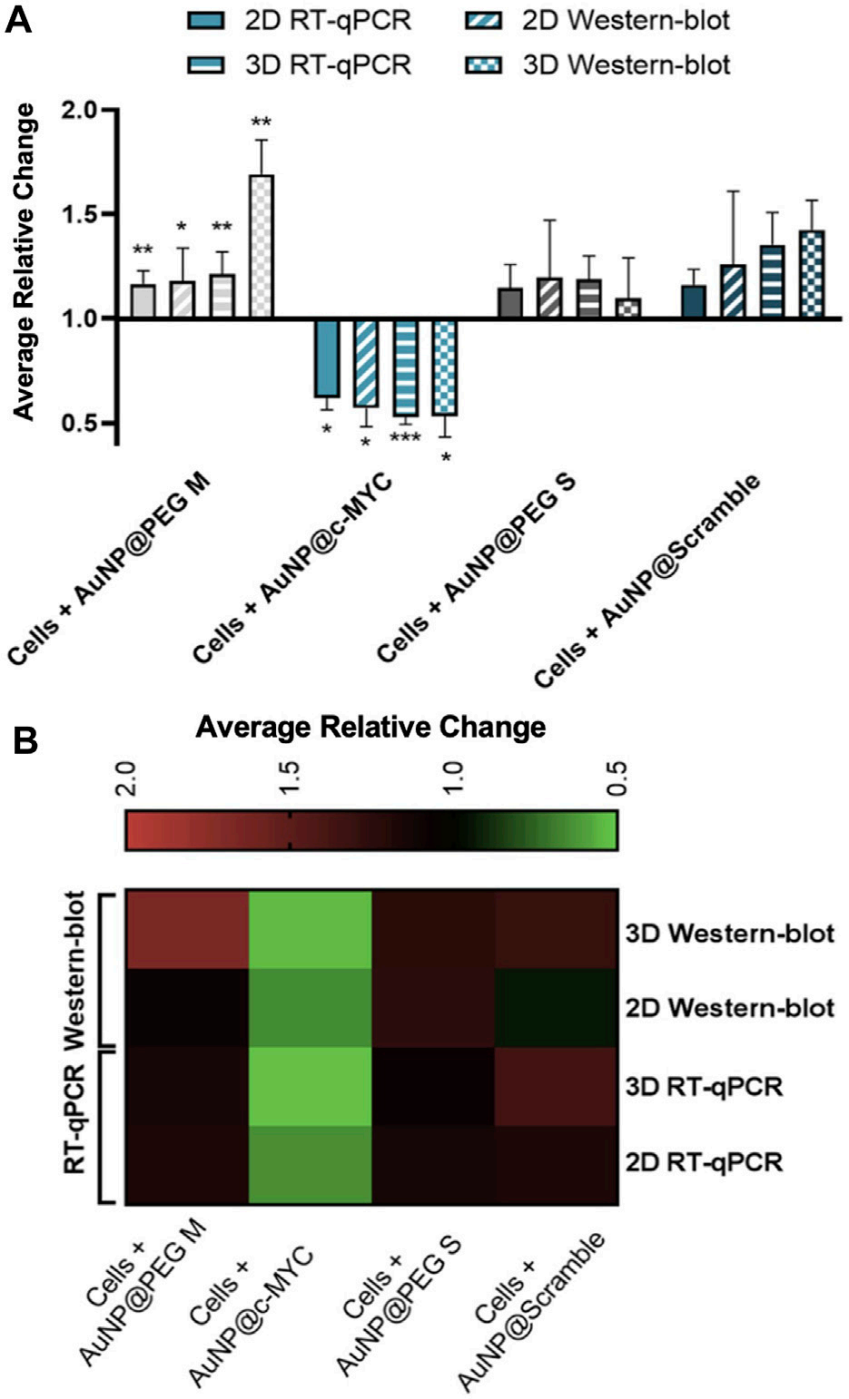
Results of c-MYC silencing on 2D and 3D cell models upon 6h of incubation with 54 nM of oligonucleotide. **(A)** Average relative change results of the c-MYC silencing of mRNA and protein in both 2D and 3D spheroids. Full bars represent the average relative change RT-qPCR values of the c-MYC silencing obtained for 2D model, stripped bars the results for 3D model, bars with diagonal stripes indicate the Western blot data of 2D model, and checkered bars for 3D model. Cells were incubated with different Au-nanoconjugates, the controls are represented by different colors: (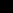) AuNP@30%PEG M, (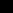) AuNP@c-MYC, (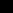) AuNP@PEG S and (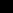) AuNP@Scramble. Bars are the result of at least 3 independent biological replicates and the error bars are the respective Standard Error Mean. Statistical analysis was performed using One-way ANOVA and Mann-Whitney test, results were considered statistically significant for *p* values <0.05. (*) represents *p* < 0.0323, (**) represents *p* < 0.0021 and (***) represents *p* < 0.0002. **(B)** Heat-map representation of the average relative change. Each column represents the different Au-oligonucleotide conjugates controls and the lines of the results obtained with Wester-blot and RT-qPCR for both 2D and 3D cell models. Values between 0.5 and 1 (gene/protein downregulation) are represented by green tones (from brighter to darker shades), values equal to 1 are represented in black, and values between 1 and 2 (gene/protein overexpression) are represented in red tones (from darker to brighter).

Overall, a similar silencing effect was obtained for mRNA and protein levels (Figure 6), in both 2D (38% vs. 43%) and 3D (43% vs. 47%) models, respectively. Studies have already reported on the decreased expression of MYC protein upon the mRNA silencing with ASOs-based therapeutics (Wickstrom et al., 1991). However, the final silencing activity might be influenced by the different uptake dynamics due to cell proliferation rate, mRNA target site, and ASOs modifications (Crooke et al., 2017). A more detailed analysis of the silencing profiles in both 2D and 3D models reveals that the silencing effect (relative change) is more marked for the 3D than for the 2D model.

However, when comparing conditions by the 2^-△△CT^ method, the opposite is observed, i.e., a silencing of 33% for the 2D-cell cultures and 20% for the 3D spheroids. Such apparent disparities might be due to *c-MYC* overexpression when spheroids are incubated with AuNP@Scramble (Figure 6B, line 3 x column 4). Since the relative change is calculated using AuNP@Scramble as a reference, the slight increase in *c-MYC* levels triggered by AuNP@Scramble, may lead to an overestimation of the silencing potential attained with AuNP@c-MYC. Since no significant increase in gene expression occurs when 2D-cells are exposed to the AuNP@Scramble (Figure 6B, Line 4 x column 4), this does not have an impact on the calculation of silencing efficacy (38% and 33% for 2^-△△CT^ method and 38% for relative change).

These data seem to indicate that cells react differently to AuNP exposure depending on whether they are cultured in 2D or 3D structures, which might be due to inherently different uptake mechanisms, ultimately causing distinct molecular responses. This is even more interesting when we consider the function of *c-MYC* on the modulation of cell proliferation and needs further investigation. When cells are grown as a monolayer on a flat surface, nanoparticles typically have direct access to the cells and can easily diffuse through the media and reach the cell membrane. Nanoparticles may then be internalized via passive diffusion or endocytosis, depending on their surface properties and the specific cellular uptake mechanisms involved (Behzadi et al., 2017). Conversely, nanoparticle uptake by 3D spheroid models has been shown to be influenced by their structure and cellular composition, including the extracellular matrix density and the barrier effect of the outer cell layers, that restricts direct access to cells in the inner layers, increasing the cellular heterogeneity and promoting oxygen and nutrient gradients (Stylianopoulos and Jain, 2015). Together, these factors alter nanoparticle diffusion rate, mobility, distribution within the spheroid and, ultimately, uptake efficiency. Understanding these differences and the way it affects the internalization rate of particles is crucial when designing experiments and interpreting results in the context of gene silencing studies (Musielak et al., 2023).

**FIGURE 7:**
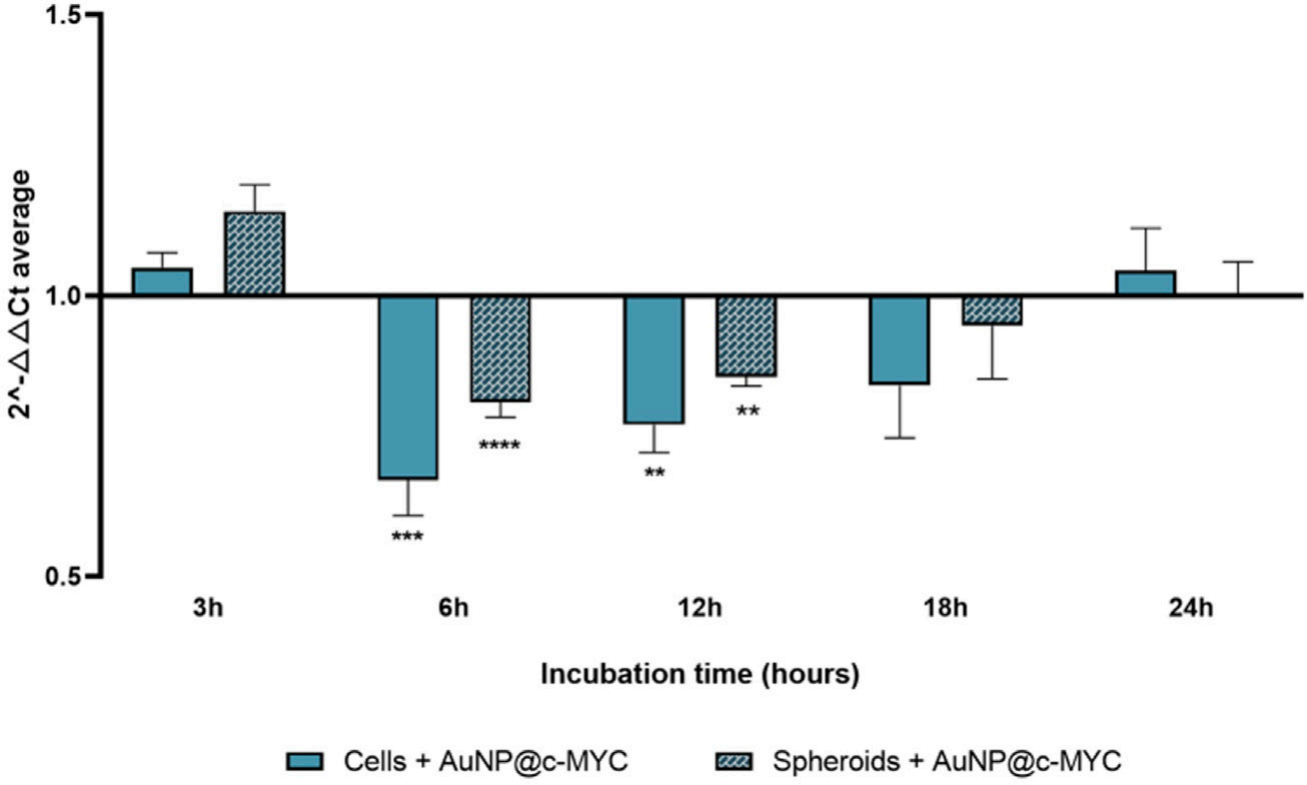
Results of *c-MYC* silencing for 6 h using 54 nM of AuNP@c-MYC on 2D and 3D cell models over different incubation times. The average 2^-△△CT^ values when using the AuNP@c-MYC nanoconjugate on 2D-cell culture is represented by (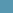) and the results for 3D-cell culture model are presented by (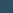). Bars are the result of at least 3 independent biological replicates and the error bars the respective Standard Error Mean. Statistical analysis was performed using One-way ANOVA and Mann-Whitney test, results were considered statistically significative for *p* values <0.05. (*) represents *p* < 0.0323, (**) represents *p* < 0.0021, (***) represents *p* < 0.0002 and (****) represents *p* ≤ 0.0001.

The time dependent silencing profiles for 54 nM of AuNP@c-MYC seem to support the hypothesis of the effect played by rate of internalization into the 3D structure in the resulting silencing capability. Both models show a similar silencing profile over time, with the maximum silencing occurring at 6 h, from which a gradual decay occurs until 18 h of incubation (minimum silencing effect), after which *c-MYC* expression returned to basal levels—Figure 7 (see also see Supplementary Figure S10).

**FIGURE 8:**
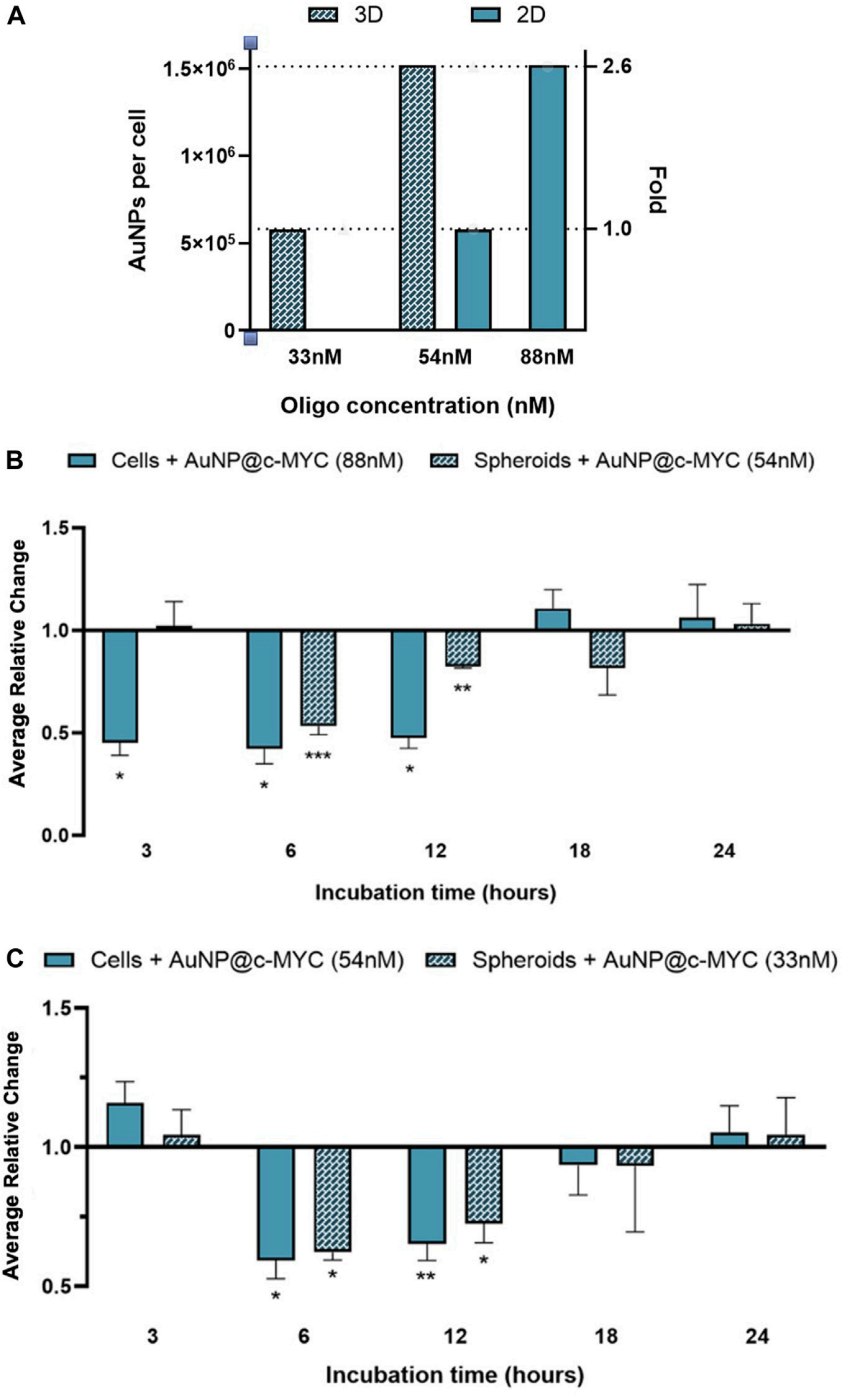
Results of *c-MYC* silencing on 2D and 3D cell models over different incubation times using the same ration of particles per cell. **(A)** Number of particles per cell for the different ASOs concentrations in each cell model. Full bars (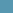) represent the 2D-cell model and checkered bars (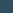) represent the spheroid model. **(B)** Relative change of the *c-MYC* silencing in both 2D-cells (88 nM of ASOs) and spheroids (54 nM of ASOs), attaining on the 2D-cell model the same ratio of particles per cell as used in spheroids. **(C)** Relative change of the *c-MYC* silencing in both 2D-cells (54 nM of ASOs) and spheroids (33 nM of ASOs), attaining on spheroids the same ratio of particles per cell as used in 2D-cell culture. The results of 2D-cell culture are represented by (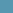) and the results for 3D-cell culture model are presented by (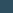). Bars are the result of at least 3 independent biological replicates and the error bars are the respective Standard Error Mean. Statistical analysis was performed using One-way ANOVA and Mann-Whitney test, results were considered statistically significant for *p* values <0.05. (*) represents *p* < 0.0323, (**) represents *p* < 0.0021 and (***) represents *p* < 0.0002.

It should be noted that, thus far the same concentration of ASO—54 nM was used to challenge cells. However, this means that each cell will be challenged with different amounts of oligo and will be exposed to a distinct number of AuNPs. Considering that a spheroid of 3 days contains around 1.9 × 10^4^ cells and each 24-well contains 2.5 × 10^5^ cells, each cell in 3D spheroids was being exposed to 2.6 times more nanoparticles than a cell grown in 2D-culture. Each cell in the spheroid was exposed to roughly 1.5 × 10^6^ particles, whereas for the same concentration of oligo (54 nM), each cell in 2D-cell culture was exposed to 5.8 × 10^5^ particles (Figure 8A). Consequently, we further calibrated the experiments to attain the same ratio of particles per cell (Figure 8; Supplementary Figure S11). To match the number of nanoparticles per cell in a spheroid corresponding to 54 nM of oligo, 2D models were exposed to 88 nM of anti-c-MYC oligo (Figure 8B). Conversely, to assess the opposite condition (spheroids with the same number of particles per cell as the one used in the 2D-cell culture), 33 nM of anti-c-MYC nanoconjugate was incubated with the spheroids (Figure 8C). As for the initial studies, these concentrations were tested for 3h, 6h, 12h, 18h, and 24 h of incubation on each cell model (see Supplementary Figures S12, S13 for silencing results with all the Au-nanoconjugates).

Exposing 2D-cultured cells to 1.5 × 10^6^ nanoparticles per cell (88 nM of oligo) showed an earlier silencing effect, starting at 3 h and lasting until 12 h after challenge, attaining an average silencing level of 54% (Figure 8B; Supplementary Figures S11A, S12). This is a far more pronounced silencing effect than that attained for the standard ratio of 5.8 × 10^5^ particles per cell (54 nM of oligo in 2D model), where noticeable silencing only occurs between 6h and 12 h of incubation, resulting on approximately 38% of silencing (Figure 8C full bars). Conversely, in 3D spheroids, the silencing effect seems to be delayed, only starting after 6 h of incubation, for the maximum silencing level (47%), decreasing to 18% at 12 and 18 h of incubation (Figure 8B). For spheroids exposed to 5.8 × 10^5^ particles per cell (33 nM of oligo), the silencing effect remains from 6 to 18 h of incubation with the Au-nanoconjugates (Figure 8C; Supplementary Figures S11B, S13). However, the silencing potential increases in comparison to the results obtained with 1.5 × 10^6^ particles per cell (54 nM), resulting on a change from 22% (54 nM) to 38% (33 nM) after 6 h of incubation, and from 15% (54 nM) to 28% (33 nM) after 12 h.

**FIGURE 9:**
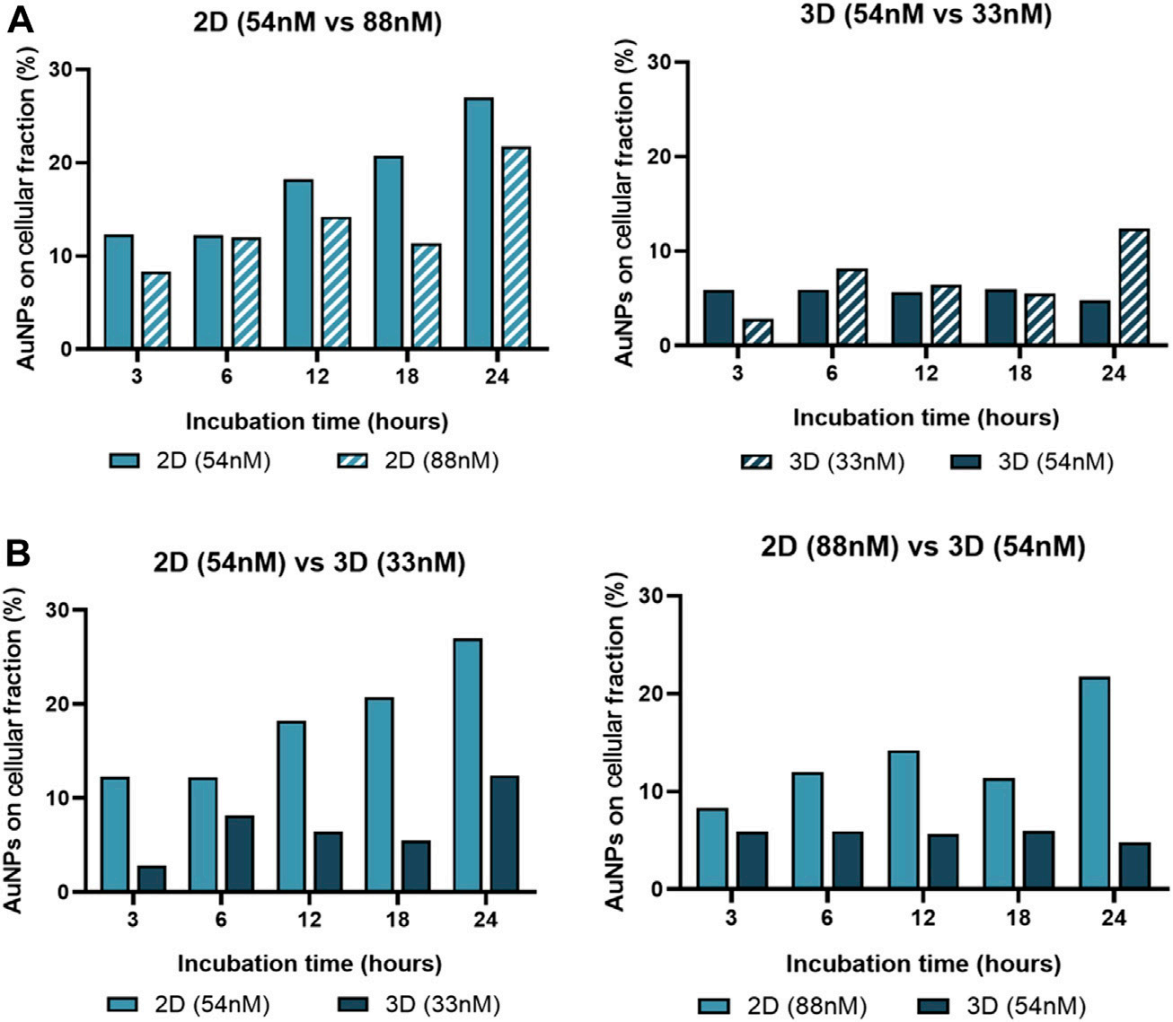
Gold content in the cell fraction. ICP-AES was used to determine the gold content on the cellular fraction of 2D cultured cells and 3D spheroids, after incubation with AuNP@c-MYC nanoconjugates for 3h, 6h, 12h, 18h and 24 h. **(A)** Percentage of Au-nanoconjugates detected in the cellular fraction after incubation with 2D cells using 54 nM (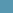) and 88 nM (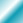) (Left) or with Spheroids using 54 nM (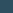) and 33 nM (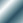) (Right). **(B)** Comparison of the percentage of Au-nanoconjugates detected in the cellular fraction 2D cells and Spheroids with the same ratio of particles per cell. Left: 5 × 10^3^ particles per cell—(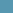) represents 2D-cultured cells with 54 nM and (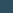) Spheroids with 33 nM. Right: 13 × 10^3^ particles per cell—(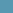) represents 2D-cultured cells with 88 nM and (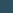) Spheroids with 54 nM.

Together, these data support the relevance of Au-nanoconjugate penetration into complex structures, which influences the number of nanoparticles per cell and, thus, the duration and efficiency of gene silencing. To further characterize this issue, the effective amount of gold in the cell fraction was determined by ICP-AES for 2D and 3D models challenged with anti-c-MYC nanoconjugate for 3h, 6h, 12h, 18h, and 24 h (Figure 9).

Data show that the average number of AuNPs in 2D cultured cells is higher when compared to cells cultured as 3D spheroids for any given ratio of initial NP/cell exposure, which is in accordance with previous studies on this matter (Belli et al., 2017). Also, the number of AuNPs on the cellular fraction of 2D-cell culture seems to increase with the incubation time, except for 18 h, this could be related with the duplication rate for HCT-116 cells (Imanis Life Sciences, 2023). Additionally, regardless of being 2D or 3D cultured cells, data show a higher uptake efficiency (on both culture schemes) with the ratio of 5.8 × 10^5^ particles per cell (33 nM of oligo on 3D and 54 nM of oligo on 2D) when compared to 1.5 × 10^6^ particles per cell (Figure 9). This phenomenon, where lower concentrations of AuNPs have better uptake than higher concentrations, has been associated with individual cellular response mechanisms and nanoparticle-cell interactions, such as saturation of cellular uptake mechanisms (Chithrani et al., 2006; Mironava et al., 2010).

Internalization of AuNPs by human cells may occur by a range of different mechanisms, among them phagocytosis, micropinocytosis, and receptor-mediated endocytosis (RME) (Conner and Schmid, 2003; Saha et al., 2013). These mechanisms use different receptors, and cellular signaling pathways and are dependent on the size and type of nanoparticles (Dobrovolskaia and Mcneil, 2007; Xie et al., 2017). AuNPs with diameters <100 nm seem to be internalized by cells mainly by receptor-mediated endocytosis, which includes caveolae-mediated, clathrin-mediated, and caveolae/clathrin-independent endocytosis (Shukla et al., 2005; Chithrani and Chan, 2007; Nativo et al., 2008). These mechanisms have a limited capacity, meaning they can only process a certain number of nanoparticles at a given time (Gao et al., 2005; Bonilla-Vidal et al., 2023). When higher concentrations of nanoparticles are present, the uptake mechanisms may become saturated, decreasing the uptake rate. Additionally, when cells are incubated with higher concentrations of particles, a higher number of particles are trying to interact with the cell membrane (lower ratio of cell surface area to AuNPs), since the surface availability of the cell membrane also dictates its wrapping effect, a lower concentration of particles (larger surface area-to-volume ratio) can result in more favorable interactions between the particles and the cell membrane, promoting higher uptake rates.

Besides, at higher concentrations, nanoparticles have a higher tendency to cluster or agglomerate, which may increase the size of nanoparticle aggregates hindering the interaction with the cell membrane and consequently reducing the effective concentration available for uptake (Chithrani et al., 2006). In contrast, lower concentrations of nanoparticles are less likely to aggregate, allowing for better dispersion and increased contact with the cell membrane, thus endorsing its uptake. The size of the particles plays a crucial role in the rate and extent of cellular uptake since it dictates the way the cellular membrane encloses the particles, the so-called “wrapping effect” (Alkilany and Murphy, 2010). It was found that nanoparticles with 27–30 nm diameter have the fastest wrapping time, and thus the fastest receptor-mediated endocytosis (Gao et al., 2005).

## 4 Conclusion

By assessing the silencing capability of the Au-nanoconjugate at the mRNA and protein levels, we were able to provide a simple yet robust tool to assist researchers in the translation of their findings from 2D to 3D cell models. Shifting to these more complex 3D models may provide for a more realistic characterization of gene silencing techniques that are closer to the observations in *in vivo* models. Using these 3D models provides for a tissue-like environment where nanoparticle-cell interactions might be tuned to adjust the silencing efficiency attained in simpler cell-culture models. The transition between 2D to 3D-cell cultures, such as spheroids, requires deeper characterization and adjustment to uptake rate, silencing efficiency, and eventual toxicity that might occur. Herein, we show how gene silencing efficacy varies with the concentration of effective ASO, nanoparticle concentration, and time point of analysis considering 2D-cells and 3D spheroids. We believe that this study provides a useful framework to assist the community in translating their findings from one type of cell model to the other. Ultimately, we hope to provide a tool to bridge the gap between *in vitro* experiments and clinical applications, offering valuable information that can guide the development of targeted therapies and personalized medicine in a more precise and clinically relevant manner.

## Data Availability

The original contributions presented in the study are included in the article/Supplementary Material, further inquiries can be directed to the corresponding author.
