# Supplementary material for: Assessing the gene silencing potential of AuNP-based approaches on conventional 2D cell culture versus 3D tumor spheroid
Source: Front Bioeng Biotechnol. 2024 Feb 12;12:1320729. doi: 10.3389/fbioe.2024.1320729 (PMC10894999; doi:10.3389/fbioe.2024.1320729)
Supplement: Supplementary file 1 [file DataSheet1.pdf]

Supplementary Material

A

	Size (nm)	PDI	Zeta Potential (mV)	Zeta Deviation	Functionalization (nr of molecules)	AuNPs' Concentration (nM)
AuNPs@Citrate	17.83	0.176	-49.1	6.12	citrate	12.40
AuNPs@30%PEG	20.74	0.175	-52.7	11.10	842 chains of PEG	58.80
AuNPs@C-Myc	36.58	0.161	-48.0	7.13	113 chains of ASOs	41.85
AuNPs@Scramble	34.28	0.168	-55.8	12.80	112 chains of ASOs	51.50

B

	Size ± SD(nm) TEM	Size ± PDI (nm) DLS	Zeta Potential ± SD (mV)	Functionalization (Nº molecules)	[AuNPs] (nM)	[ASOS] (uM)
AuNPs@Citrate	12 ± 1	17.83 ± 0.18	-49.1 ± 6.12	citrate	12.40	-
AuNPs@30%PEG	12 ± 1	20.74 ± 0.18	-52.7 ± 11.10	842 chains of PEG	58.80	-
AuNPs@C-Myc	12 ± 1	36.58 ± 0.16	-48.0 ± 7.13	113 chains of ASOs	41.85	5.55
AuNPs@Scramble	12 ± 1	34.28 ± 0.17	-55.8 ± 12.80	112 chains of ASOs	51.50	5.99

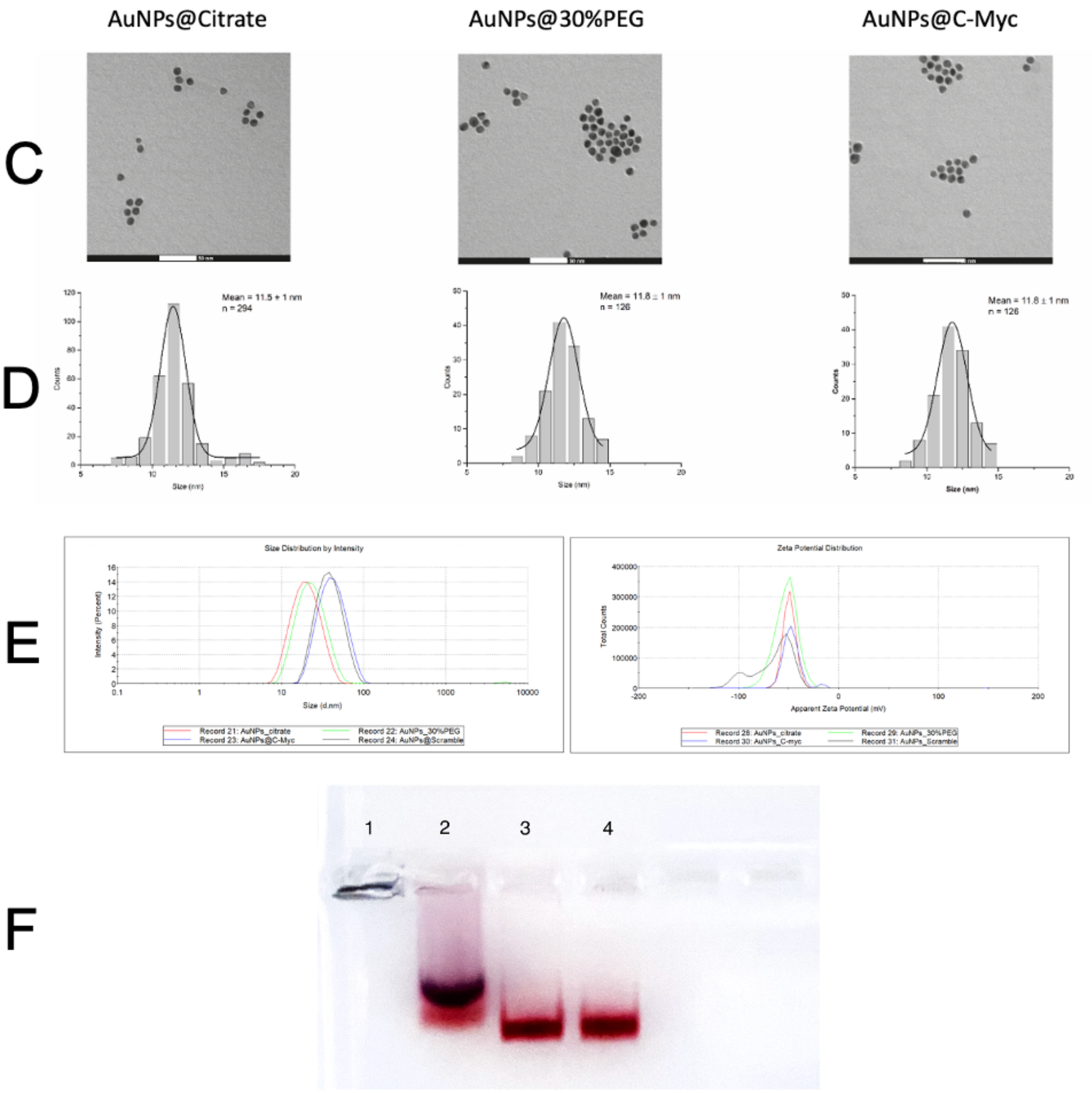

Supplementary Figure S 1. Au-oligonucleotide conjugates characterization. (A) Characterization data of all Au-oligonucleotide conjugates, regarding size by DLS and TEM, Zeta potential, number of

molecules functionalized in the particle' surface, stock concentration, and ASO concentration. **(B)** Representative transmission electron micrographs (TEM) of AuNPs@Citrate, AuNPs@30%PEG and AuNP@c-MYC and **(C)** the corresponding histograms showing the size distributions of determined from transmission electron micrographs using a Tecnai Spirit transmission electron microscope (TEM; FEI Technai G2 Spirit, Thermo Fisher Scientific, Waltham, MA, USA) equipped with a Veleta CDD camera (Veleta, Olympus, Tokyo, Japan). **(D)** Representative data of hydrodynamic size distribution (Left) and Zeta Potential distribution (Right). (Malvern Zetasizer Nano ZS at 37°C, scattering angle 173°, and laser wavelength 633 nm). **(F)** Agarose gel electrophoresis of AuNPs**.** Electrophoresis was performed using a 0.5% agarose gel and run at 70mV for 40 minutes. Each lane contains 15uL of the respective AuNPs solution and 25uL of glycerol 30% (used as loading buffer). Legend: Lane 1. AuNP@Citrate; Lane 2. AuNP@30%PEG; Lane 3. AuNP@c-MYC; Lane 4. AuNP@Scramble.

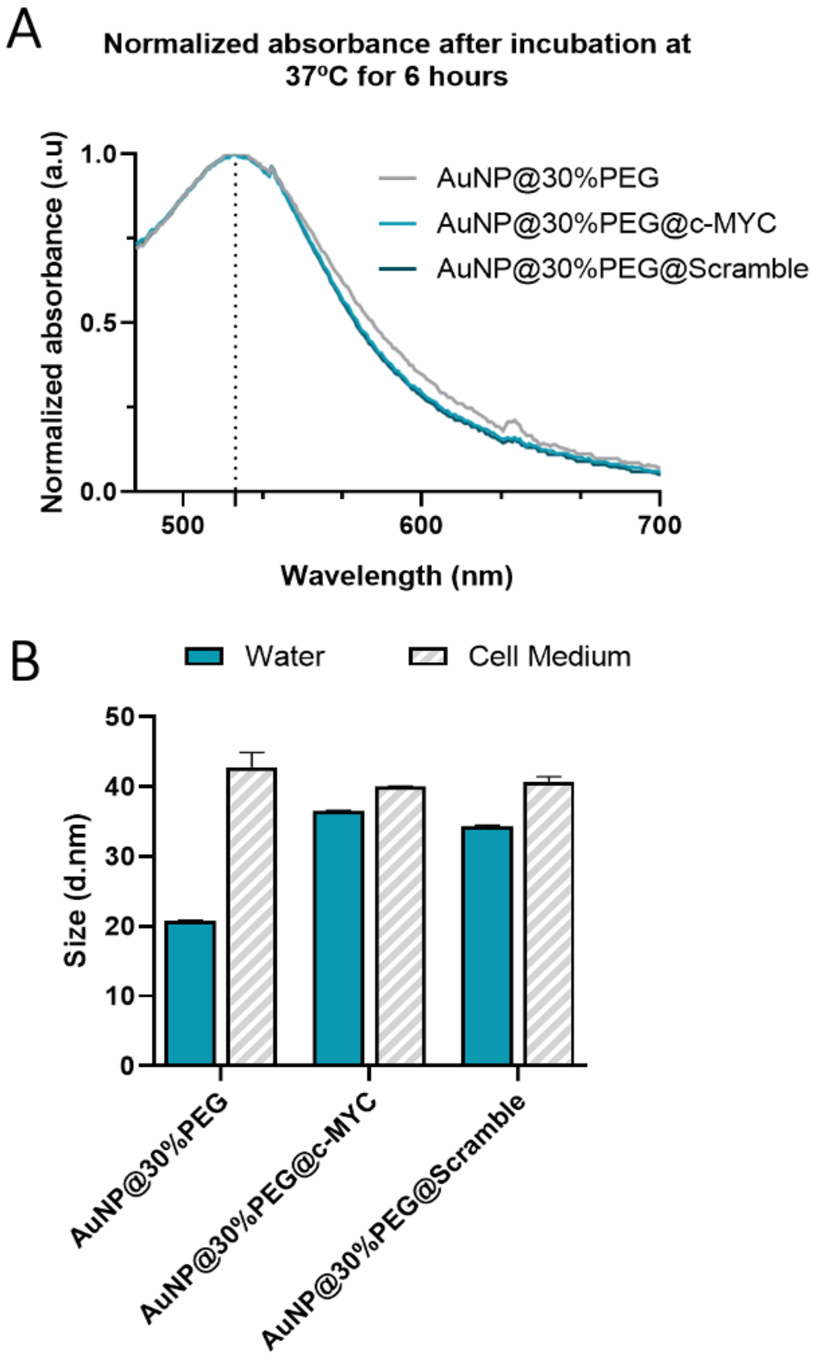

Supplementary Figure S 2. Au-oligonucleotide conjugates stability characterization after incubation at 37ºC for 6 hours. (A). Normalized UV-vis spectroscopy results after incubation with cell medium at 37ºC for 6 hours. All nanoconjugates shown the maximum peak correspondent to the LSPR peak at the 522 ± 2 nm (represented by the dotted line). **(B)** Size results attained with Dynamic Light Scattering results for the different nanoconjugates after incubation with cell medium (■) in comparison with each nanoconjugate in water (■).

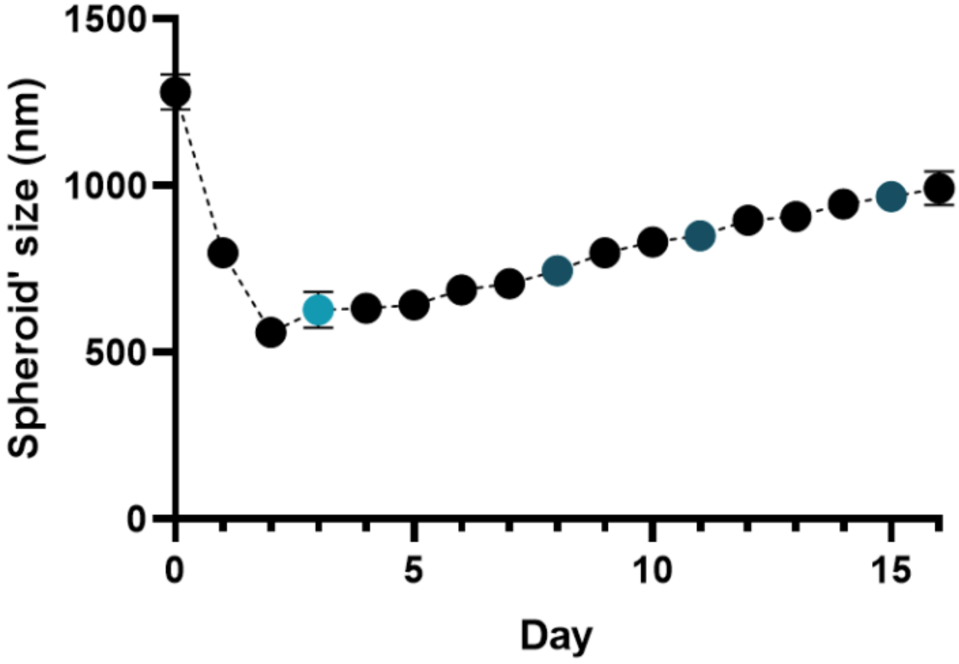

Supplementary Figure S 3. Size of HCT-116 spheroids over the days. The results are an average of the Ferret's diameter of 10 independent spheroids. The error bars are the respective standard deviation. The measurements were performed on microscope images acquired using Ti-U eclipse inverted microscope (Nikon, Tokyo, Japan) and further analyzed on Image J software. (●) represents the average size of a spheroid at 3 days of growth (condition used for the gene silencing assays) and (●) represents the spheroid' size on the days where cell culture medium was exchanged to allow continuous growth.

Supplementary Information S4

$$(Eq\ 1) Realative\ change\ PEG\ M = \frac{2^{-\Delta\Delta \mathrm{CT}}\ (PEG\ M)}{2^{-\Delta\Delta \mathrm{CT}}\ (Cells)}$$

$$(Eq\ 2) Realative\ change\ PEG\ S = \frac{2^{-\Delta\Delta \mathrm{CT}}\ (PEG\ S)}{2^{-\Delta\Delta \mathrm{CT}}\ (Cells)}$$

$$(Eq\ 3) Realative\ change\ Scramble = \frac{2^{-\Delta\Delta \mathrm{CT}}\ (Scramble)}{2^{-\Delta\Delta \mathrm{CT}}\ (PEG\ S)}$$

$$(Eq\ 4) Realative\ change\ c - MYC = \frac{2^{-\Delta\Delta \mathrm{CT}}\ (c - MYC)}{2^{-\Delta\Delta \mathrm{CT}}\ (Scramble)}$$

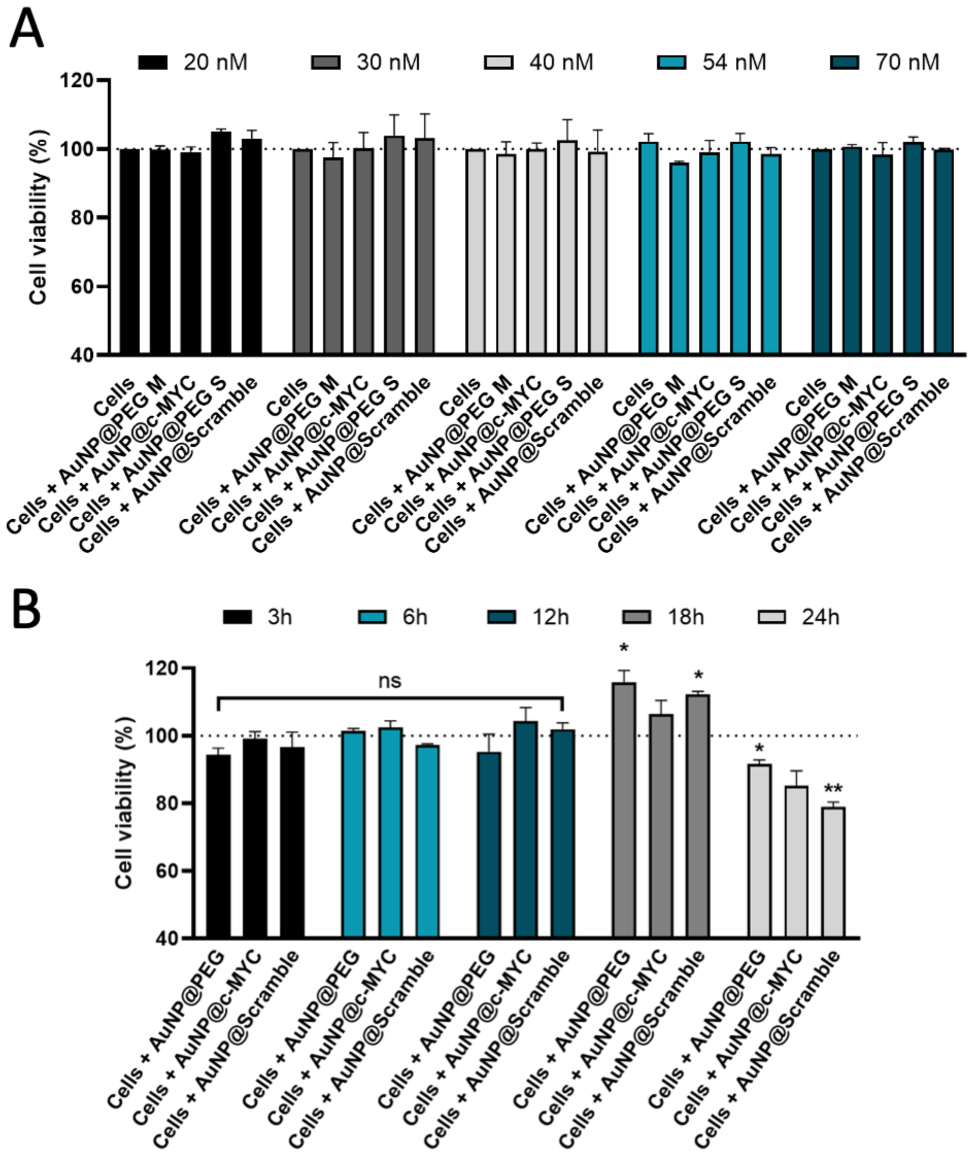

Supplementary Figure S 4. MTS assay results after the incubation of cells with different concentrations and challenge time-points with each nanoconjugate. (A) MTS assay results after 6 hours of challenge with each nanoconjugate. Bars represent the normalized cell viability to the "Cells" control, for the incubation with Au-oligonucleotide conjugate at a concentration of 20nM (■), 30nM (■), 40nM (■), 54nM (■) and 70nM (■). **(B)** MTS assay results for cell incubation with 54nM of Au-nanoconjugates after 3, 6, 12, 18 and 24 hours of challenge. Bars represent the normalized cell viability to the "Cells" control, for the incubation with each Au-oligonucleotide conjugate after an incubation period of 3h (■), 6h (■), 12h (■). 18h (■) and 24 h (■). Bars are the result of 3 independent biological replicates with 2 technical replicates, and the error bars the respective Standard deviation. Statistical analysis was performed to assess cell viability differences in relation to "Cells control" represented by the dotted line at 100%, using One-way ANOVA, the results were considered statistically significative for p values < 0.05. (*) represents $p \leq 0.0323$, (**) represents $p \leq 0.0021$.

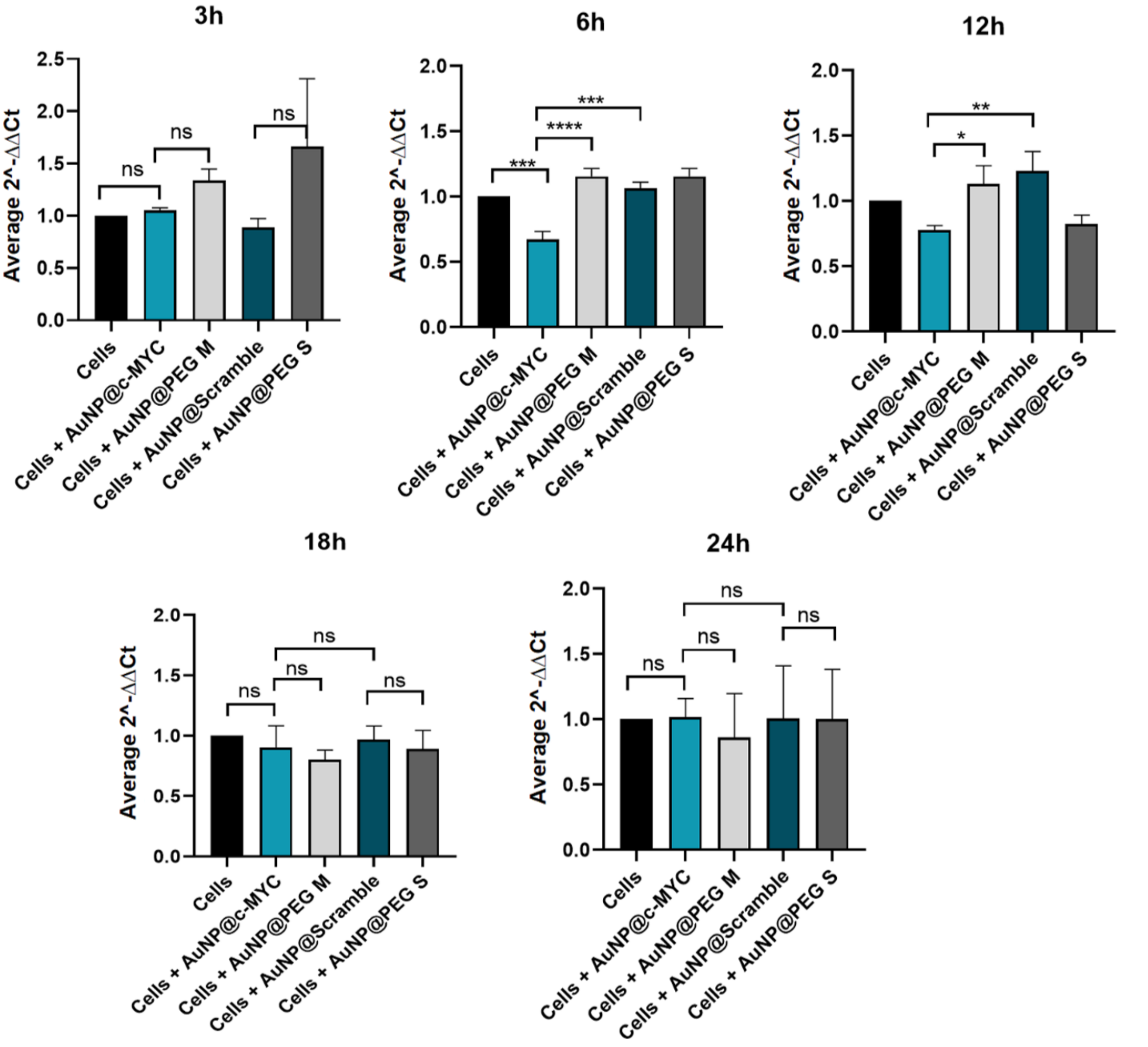

Supplementary Figure S 5. $2^{-\Delta\Delta CT}$ results of *c-MYC* silencing for 3, 6, 12, 18 and 24 hours with Au-nanoconjugates incubation with cells, using 54nM of concentration. Full bars represent the average result of 3 biological replicates, for the cells with the incubation with only medium (■), AuNP@c-MYC (■), AuNP@PEG M (■), AuNP@Scramble (■) and AuNP@PEG S (■) for either 3h, 6h, 12h, 18h and 24h. Error bars represent the Standard Error Mean. Statistical analysis was performed using One-way ANOVA and Mann-Whitney test, results were considered statistically significative for p values < 0.05. (*) represents $p \leq 0.0323$, (**) represents $p \leq 0.0021$, (***) represents $p \leq 0.0002$ and (****) represents $p < 0.0001$.

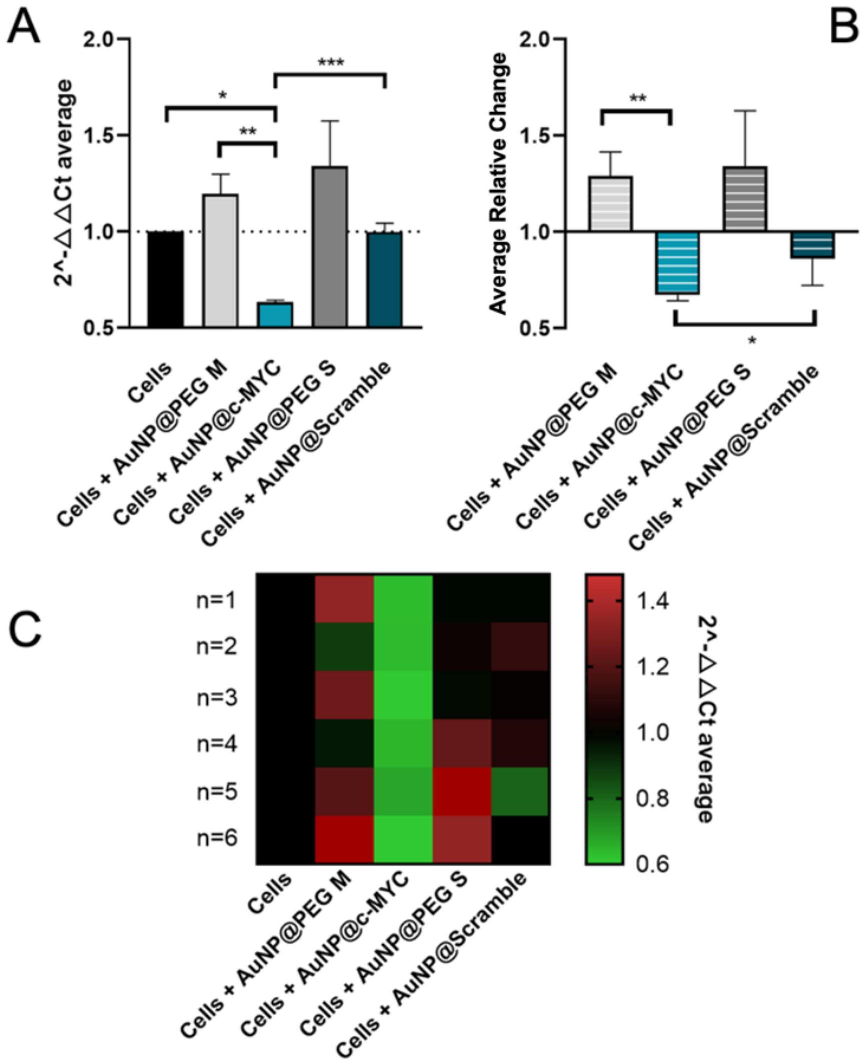

Supplementary Figure S 6. Results of *c-MYC* silencing using 54nM of Au-nanoconjugates for 6 hours of challenge time. (A) Average $2^{-\Delta\Delta CT}$ results and **(B)** Average relative change of *c-MYC* silencing. Full bars represent the $2^{-\Delta\Delta CT}$ and striped bars the relative change for each condition controls: (■) cells incubated with medium only, (■) AuNPs@30%PEG M, (■) AuNP@c-MYC, (■) AuNPs@PEG S and (■) AuNPs@Scramble. Bars are the result of 6 independent biological replicates and the error bars the respective Standard Error Mean. **(C)** Heat-map representation of the average $2^{-\Delta\Delta CT}$ results of *c-MYC* silencing. Each column represents the different Au-oligonucleotide conjugates controls, and the lines the results of each biological replicate. $2^{-\Delta\Delta CT}$ values between 0.6 and 1 (gene silencing) are represented by green tones (from brighter to darker shades), $2^{-\Delta\Delta CT}$ values equal to 1 are represented in black and values between 1 and 1.5 (gene overexpression) are represented in red tones (from darker to brighter). Statistical analysis was performed using One-way ANOVA and Mann-Whitney test, results were considered statistically significative for p values < 0.05. (*) represents $p \leq 0.0323$, (**) represents $p \leq 0.0021$ and (***) represents $p \leq 0.0002$.

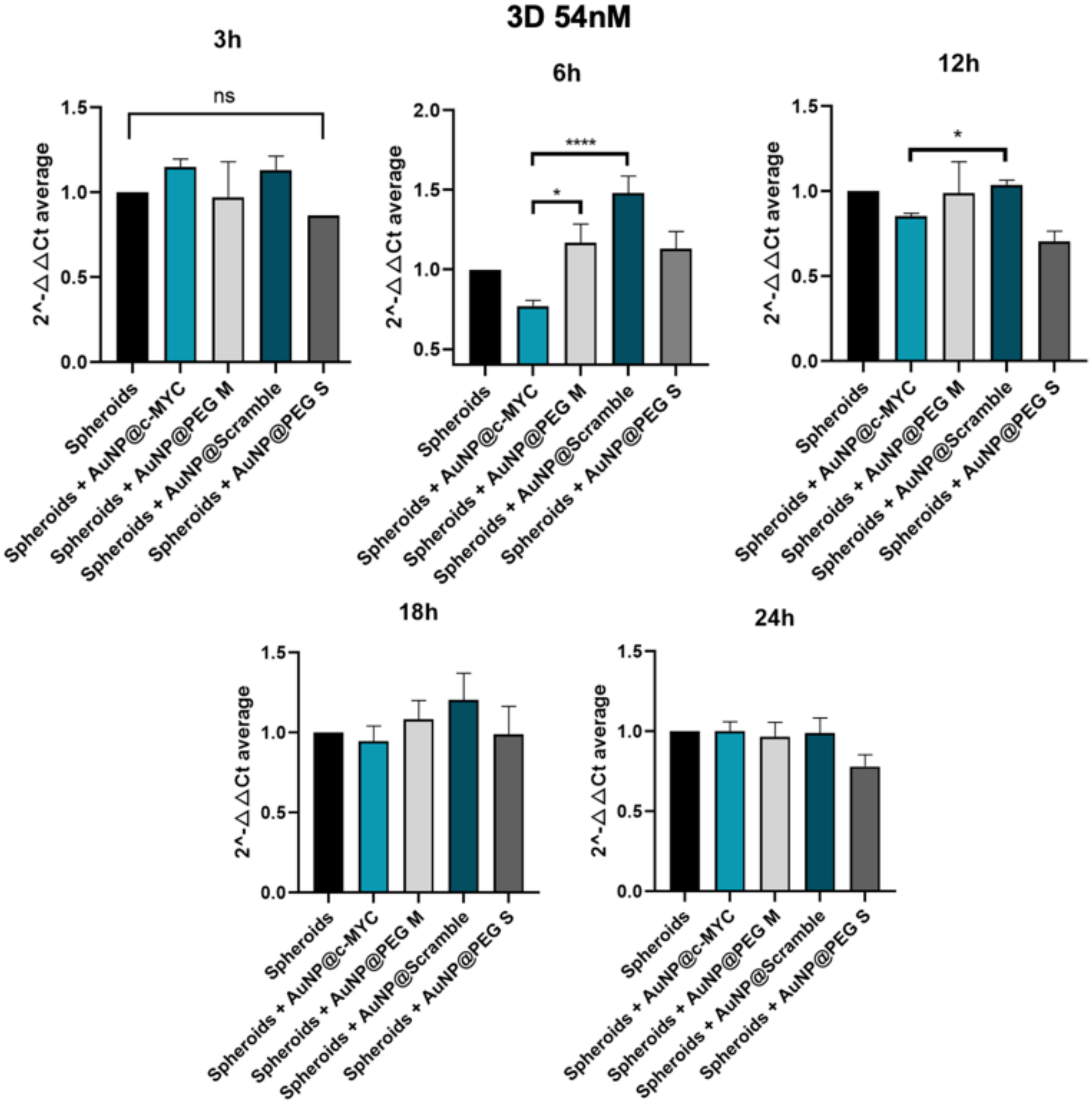

Supplementary Figure S 7. $2^{-\Delta\Delta CT}$ results of spheroid culture challenge with 54nM of Au-oligonucleotide conjugates at different incubation periods. Bars are the result of at least 3 independent biological replicates and the error bars the respective Standard Error Mean. Statistical analysis was performed using One-way ANOVA and Mann-Whitney test, results were considered statistically significative for p values < 0.05. (*) represents $p \leq 0.0323$, (**) represents $p \leq 0.0021$, (***) represents $p \leq 0.0002$ and (****) represents $p < 0.0001$.

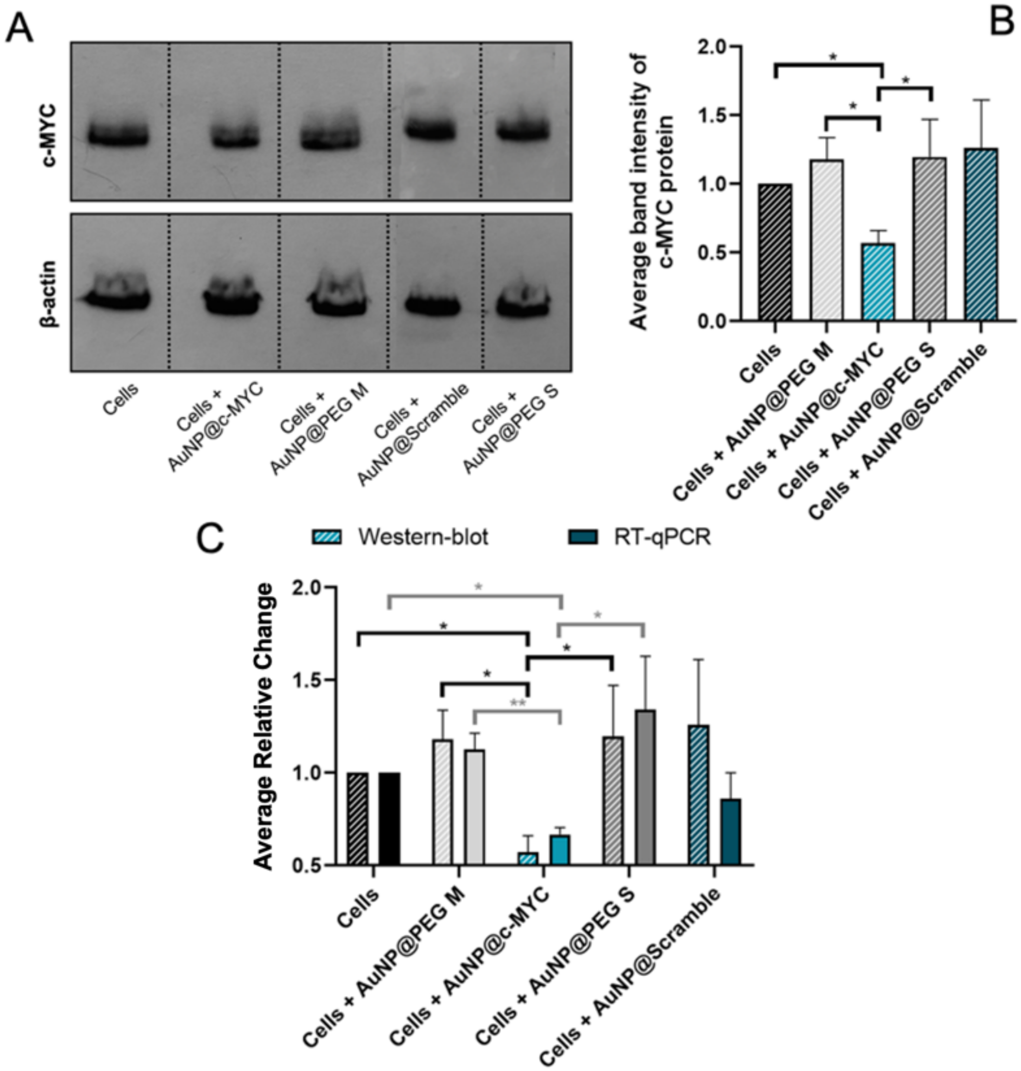

Supplementary Figure S 8. Results of *c-MYC* silencing at protein level using 54nM of Au-nanoconjugates for 6 hours of challenge time. (A) Representative image of a Western-blot membrane for the detection of C-MYC (top line) and β-ACTIN (bottom line) proteins, after incubation with the respective Au-oligonucleotide conjugates using the previously optimized conditions. **(B)** Average results for the band intensity obtained for C-MYC protein (normalized to the respective β-Actin control) in 3 independent Western-blot experiments. **(C)** Average relative change results of *c-MYC* silencing obtained on mRNA level by RT-qPCR and protein level by Western-blot. Striped bars represent the results of Western-blot and full bars the results of RT-qPCR, for each experiment controls: (■) cells incubated with medium only, (■) AuNPs@30%PEG M, (■) AuNP@c-MYC, (■) AuNPs@PEG S and (■) AuNPs@Scramble. Bars are the result of at least 3 independent biological replicates, and the error bars the respective Standard Error Mean. Statistical analysis was performed using One-way ANOVA and Mann-Whitney test, results were considered statistically significative for p values < 0.05. (*) represents $p \leq 0.0323$ and (**) represents $p \leq 0.0021$.

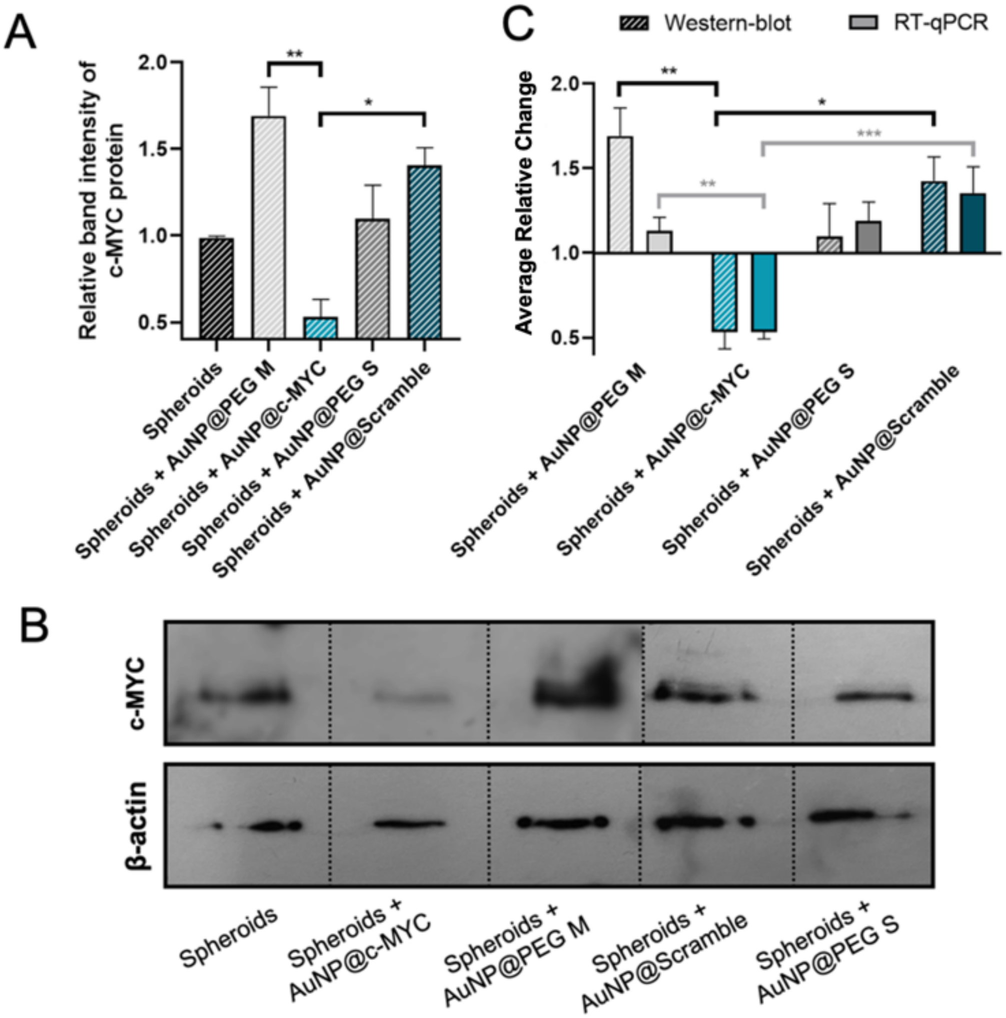

Supplementary Figure S 9. Results of silencing c-MYC protein on spheroids upon 6h of incubation with 54nM of oligonucleotide. (A) Average results for the band intensity obtained for C-MYC protein (normalized to the respective β-Actin control) in 3 independent Western-blot experiments. **(B)** Representative image of a Western-blot membrane for the detection of C-MYC (top line) and β-ACTIN (bottom line) proteins, after incubation with the respective Au-oligonucleotide conjugates. **(C)** Average relative change of *c-MYC* silencing obtained on mRNA level by RT-qPCR and protein level by Western-blot. Striped bars represent the results of Western-blot and full bars the results of RT-qPCR, for each experiment controls: (■) spheroids incubated with medium only, (■) AuNPs@30%PEG M, (■) AuNP@c-MYC, (■) AuNPs@PEG S and (■) AuNPs@Scramble. Bars are the result of at least 3 independent biological replicates, and the error bars the respective Standard Error Mean. Statistical analysis was performed using One-way ANOVA and Mann-Whitney test, results were considered statistically significative for p values < 0.05. (*) represents $p \leq 0.0323$, (**) represents $p \leq 0.0021$ and (***) represents $p \leq 0.0002$.

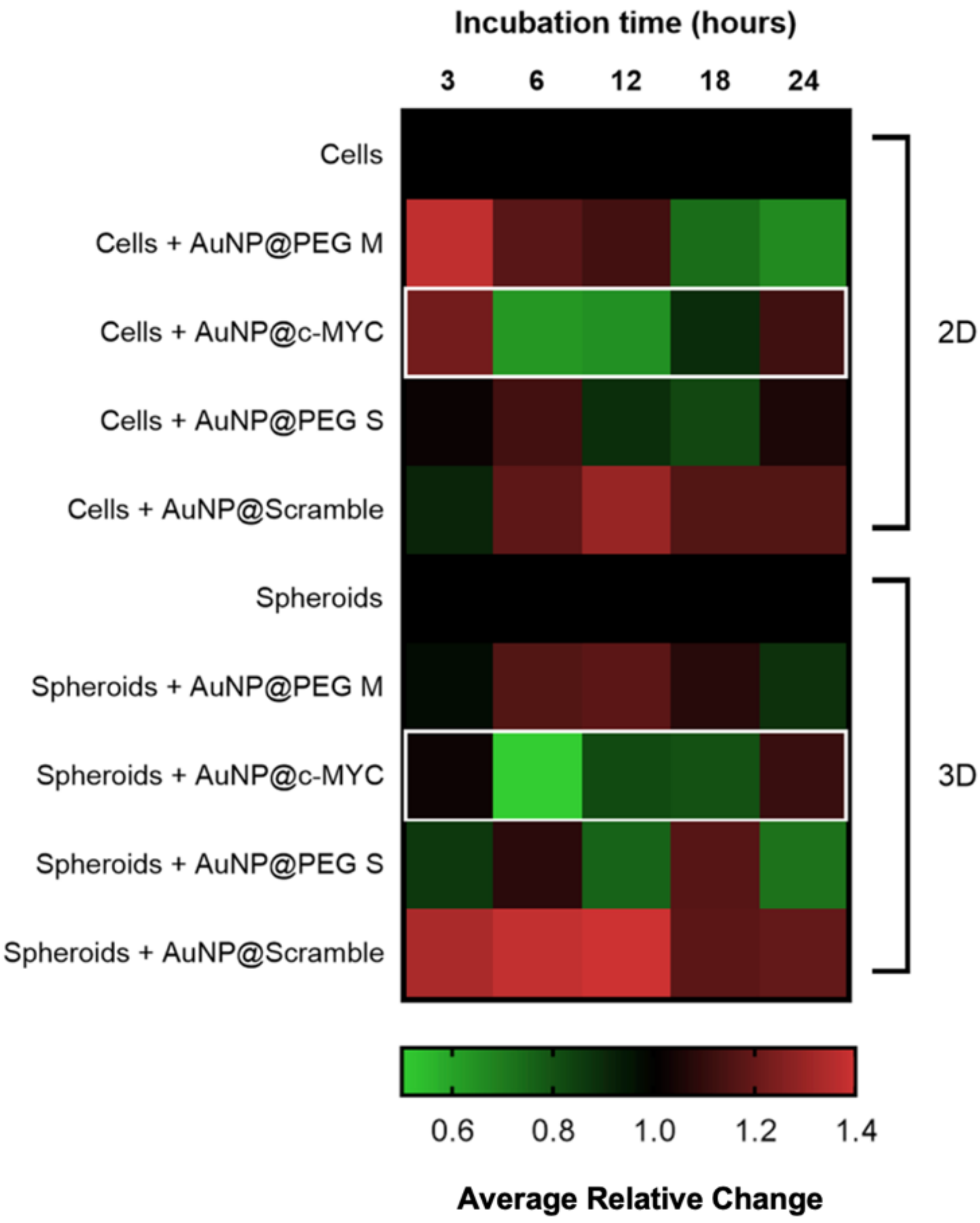

Supplementary Figure S 10. Results of *c-MYC* silencing using 54nM of oligonucleotide on 2D and 3D cell models over different incubation times. Heat-map representation of the average relative change for the incubation for 3h, 6h, 12h, 18h and 24h with 54nM of each Au-nanoconjugate in 2D and 3D cell models. Each column represents a different incubation time, and the lines the results obtained for each Au-oligonucleotide conjugate. Values between 0.5 and 1 (gene/protein downregulation) are represented by green tones (from brighter to darker shades), values equal to 1 are represented in black and values between 1 and 1.4 (gene/protein overexpression) are represented in red tones (from darker to brighter).

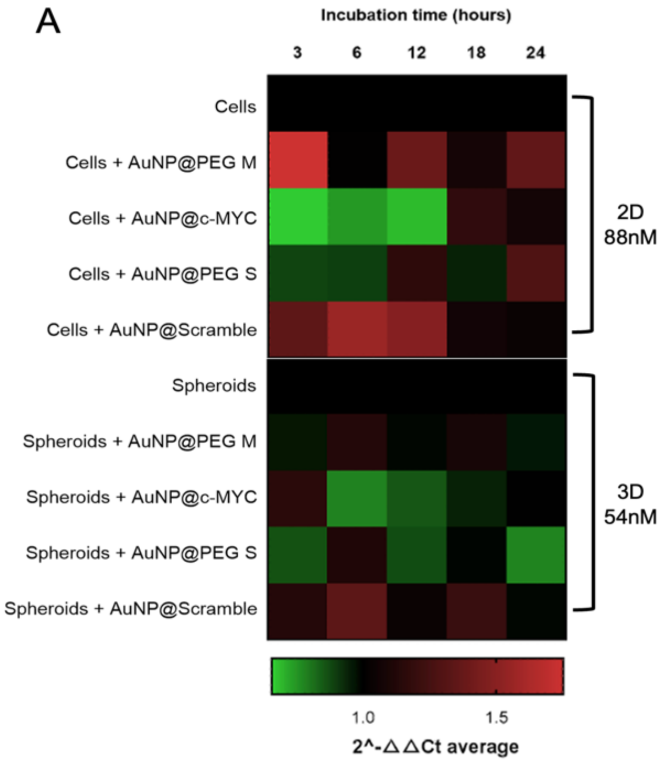

Supplementary Figure S 11. $2^{-\Delta\Delta CT}$ results of *c-MYC* silencing on 2D and 3D cell models for the same ratio of particles per cell, over different incubation times. (A) Heat-map representation of the average $2^{-\Delta\Delta CT}$ results when adjusting the concentration of Au-nanoconjugates aiming for the same ratio of particles per cell as in typical spheroid conditions (2D – 88nM vs 3D – 54nM) and **(B)** in typical 2D conditions (2D – 54nM vs 3D – 33nM). Each column represents a different incubation time, and the lines the results obtained for each Au-oligonucleotide control. $2^{-\Delta\Delta CT}$ between 0.5 and 1 (gene/protein downregulation) are represented by green tones (from brighter to darker shades), values equal to 1 are represented in black and values between 1 and 2 (gene/protein overexpression) are represented in red tones (from darker to brighter).

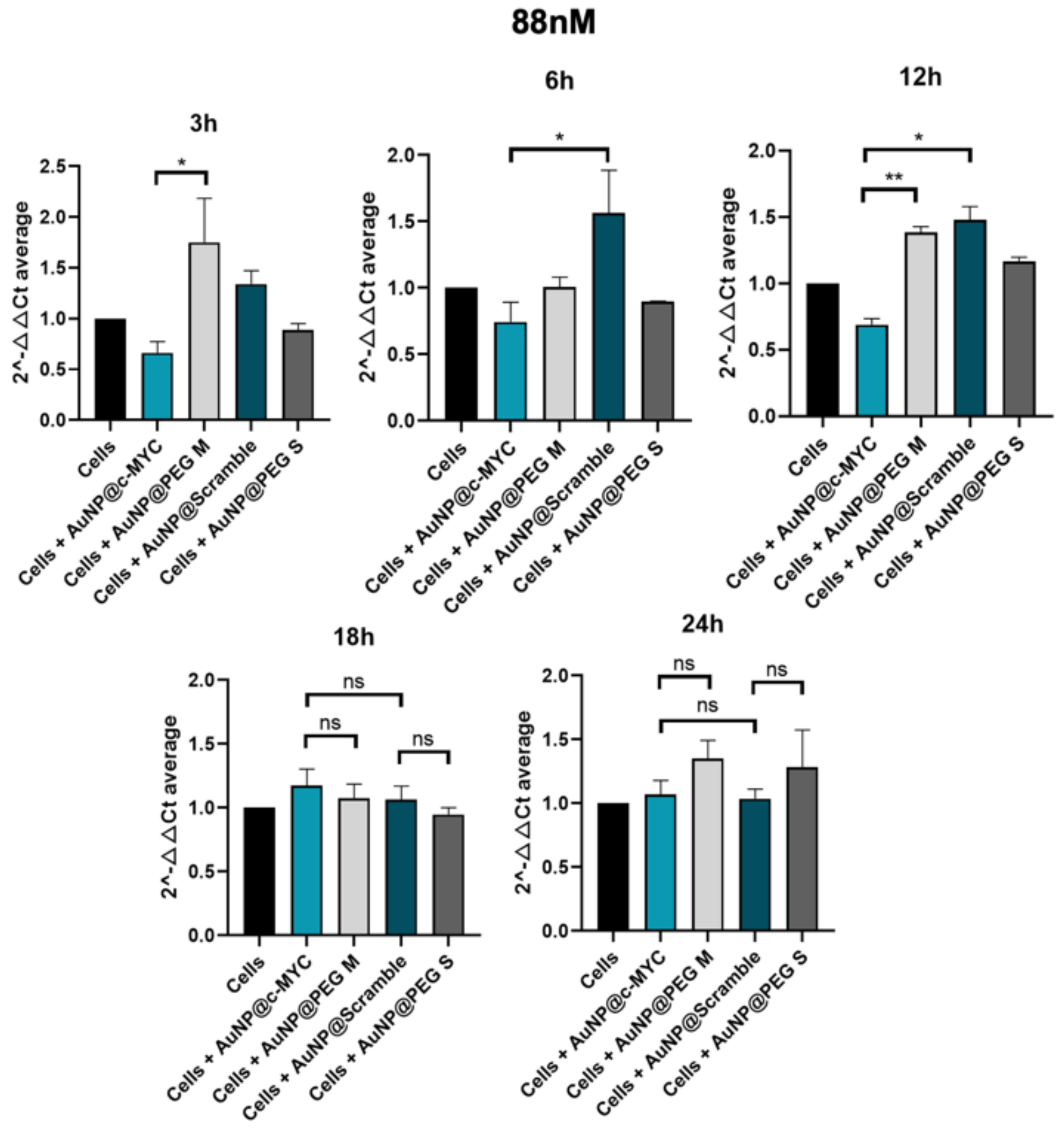

Supplementary Figure S 12. $2^{-\Delta\Delta CT}$ results of 2D cell culture challenge with 88nM of Au-oligonucleotide conjugates at different incubation periods. Bars are the result of at least 2 independent biological replicates and the error bars the respective Standard Error Mean. Statistical analysis was performed using One-way ANOVA and Mann-Whitney test, results were considered statistically significative for p values < 0.05. (*) represents $p \leq 0.0323$ and (**) represents $p \leq 0.0021$.

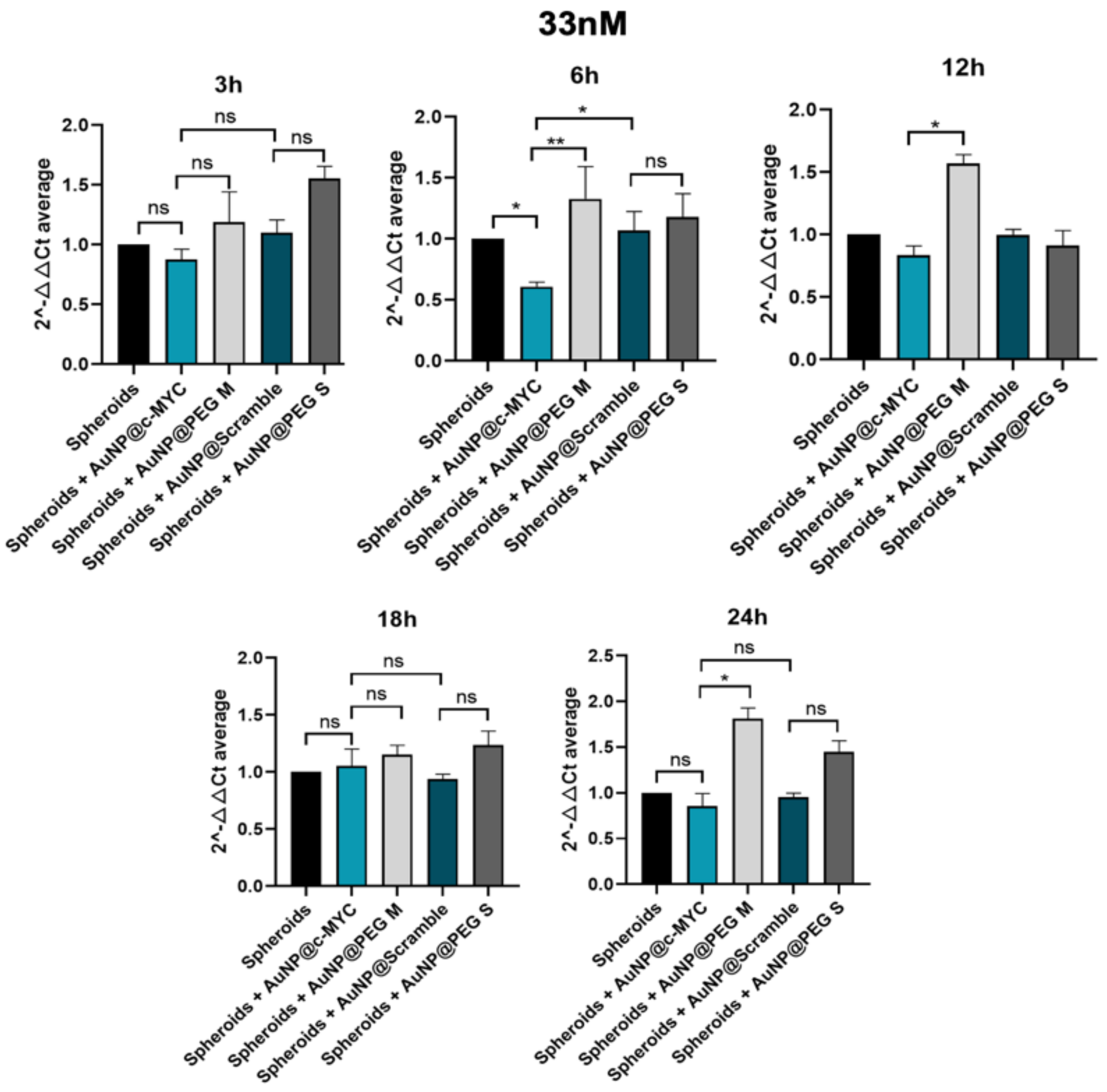

Supplementary Figure S 13. $2^{-\Delta\Delta CT}$ results of spheroid culture challenge with 33nM of Au-oligonucleotide conjugates at different incubation periods. Bars are the result of at least 2 independent biological replicates and the error bars the respective Standard Error Mean. Statistical analysis was performed using One-way ANOVA and Mann-Whitney test, results were considered statistically significative for p values < 0.05. (*) represents $p \leq 0.0323$ and (**) represents $p \leq 0.0021$.
